# Control of organelle gene expression by the mitochondrial transcription termination factor mTERF22 in *Arabidopsis thaliana* plants

**DOI:** 10.1371/journal.pone.0201631

**Published:** 2018-07-30

**Authors:** Sofia Shevtsov, Keren Nevo-Dinur, Lior Faigon, Laure D. Sultan, Michal Zmudjak, Mark Markovits, Oren Ostersetzer-Biran

**Affiliations:** Department of Plant and Environmental Sciences, The Alexander Silberman Institute of Life Sciences, The Hebrew University of Jerusalem, Givat-Ram, Jerusalem, Israel; Universidade Federal de Vicosa, BRAZIL

## Abstract

Mitochondria are key sites for cellular energy metabolism and are essential to cell survival. As descendants of eubacterial symbionts (specifically α-proteobacteria), mitochondria contain their own genomes (mtDNAs), RNAs and ribosomes. Plants need to coordinate their energy demands during particular growth and developmental stages. The regulation of mtDNA expression is critical for controlling the oxidative phosphorylation capacity in response to physiological or environmental signals. The mitochondrial transcription termination factor (mTERF) family has recently emerged as a central player in mitochondrial gene expression in various eukaryotes. Interestingly, the number of mTERFs has been greatly expanded in the nuclear genomes of plants, with more than 30 members in different angiosperms. The majority of the annotated mTERFs in plants are predicted to be plastid- or mitochondria-localized. These are therefore expected to play important roles in organellar gene expression in angiosperms. Yet, functions have been assigned to only a small fraction of these factors in plants. Here, we report the characterization of mTERF22 (At5g64950) which functions in the regulation of mtDNA transcription in *Arabidopsis thaliana*. GFP localization assays indicate that mTERF22 resides within the mitochondria. Disruption of mTERF22 function results in reduced mtRNA accumulation and altered organelle biogenesis. Transcriptomic and run-on experiments suggest that the phenotypes of *mterf22* mutants are attributable, at least in part, to altered mitochondria transcription, and indicate that mTERF22 affects the expression of numerous mitochondrial genes in Arabidopsis plants.

## Introduction

Mitochondria have their own intrinsic genetic system (the mtDNA) which is related to prokaryotes (i.e., circular DNAs, transcription of polycistronic RNAs, and the translation organellar-encoded proteins by prokaryotic-type ribosomes). Although all mitochondria likely evolved from a common eubacterial ancestor, notable genomic rearrangements have occurred in the mtDNAs of different eukaryotic lineages [1]. In animals the mtDNAs are normally small (i.e., 16~19 kbp), encoding 37 or fewer tightly packed genes. Yet, the mtDNAs in plants are notably larger (between 70 to 11,000 kb in size [2, 3]) and more variable in size and structure (reviewed by [3–7]). In angiosperms, the mtDNAs contains about 60 identifiable genes, encoding tRNAs, rRNAs, ribosomal proteins and various subunits of the energy transduction pathway, but also harbor a huge number of genes encoding open-reading-frames (ORFs) with as yet unknown functions [see e.g., [7]].

The coordination of growth and development is achieved by cellular signaling cascades, which allow plants to regulate the energy demands during particular growth and developmental stages. These involve both anterograde (nucleus-to-organelle) and retrograde (organelle-to-nucleus) signaling [8], but the identity of the messenger molecules involved in these complex pathways remains elusive. Accumulating data suggest that the expression of the mtDNA in plants is regulated mainly at the posttranscriptional level (reviewed by e.g., [9]). This is reflected by their complex RNA metabolism, the extended half-lives of many mitochondrial transcripts (mtRNAs), and the fact that the translation of various mitochondrial proteins seems uncoupled from the transcription [9]. Proteomic studies further indicate the presence of exceptionally large number of proteins containing either DNA- or RNA-binding motifs in plants mitochondria [10–13]. These factors are expected to play key roles in mtDNA expression, and may represent instruments of the nuclear control of organellar biogenesis in plants. Our work focuses on mTERF22, a member of the mitochondrial transcription termination factor (mTERF) family in Arabidopsis plants.

In animals, the transcription of mitochondrial genes is typically initiated by two loci, found in opposite directions within the ‘displacement’ (D-loop) region, which control the synthesis of two large polycistronic transcripts that span the entire organellar genome [14]. The primary transcripts (pre-RNAs) are then processed to form individual mRNAs, which can be translated by the organellar ribosomes. The physical arrangement of the genes in the mDNAs of plants is notably different [9, 15, 16]. The transcription of the complete set of genes in plants mitochondria involves numerous transcription-initiation loci [17], a situation which may have arose in plants as a consequence of the frequent rearrangements in the mtDNAs of land-plants [5–7, 18]. The identity and characterization of factors which regulate the transcription of mitochondrial genes in plants is under investigation. Here we report that mTERF22 is the first mTERF factor having a role in plant mitochondrial transcription.

In animals, the mitochondrial transcription machinery is composed of a single phage-type RNA polymerase (i.e., RpoT) and two accessory factors, denoted as mTFA and mTFB [19]. In addition to mTFA and mTFB, the expression of several mitochondrial genes also relies on members of the mitochondrial transcription termination factor (mTERF) family [20]. In vertebrates these include three or four mTERFs, which function in mtDNA transcription and ribosome biogenesis [19–21]. Angiosperms encode three different RpoT-related polymerases [22]: these including a plastidial and a mitochondrial isoforms (i.e., RpoTp and RpoTm, respectively), and in some cases an additional member, termed as RpoTmp, that is localized to both these organelles. Published data indicate that RpoTm serves as the main mtRNA polymerase, whereas RpoTmp seems to control the expression of several respiratory subunits under specific developmental stages [23–25]. Plants are lacking functional homologs of mTFA or mTFB [26], but harbor numerous genes that contain the conserved ~30 amino acids MTERF motif [27–32]. Many of the plant mTERFs are postulated to reside within the mitochondria or chloroplasts (Table 1 and [28]), and are therefore expected to play important roles in organellar gene expression. Still, there is only a limited mechanistic information regarding to the roles of these factors in plants.

**Table 1:** The mTERF family in *Arabidopsis thaliana* (ecotype Col-0).

Gene annotations*^1^	accession number	Intracellular locations*^2^	mutants phenotype / protein function	References
mTERF1 (EMB93, SOLDAT10)	AT2G03050	chloroplasts	embryo arrest	[33]
mTERF2 (EMB2219)	AT2G21710	chloroplasts	embryo arrest	[34]
mTERF3	AT2G36000	chloroplasts	no obvious phenotype	
mTERF4 (BSM/RUG2)	AT4G02990	chloroplasts	embryo arrest / group II introns splicing	[35–37]
mTERF5	AT4G14605	chloroplasts	defective chloroplast development	[38]
mTERF6	AT4G38160	mitochondria	impairment of chloroplast development / seedling lethality / tRNA^Ile^ (GAU) maturation	[39, 40]
mTERF7	AT5G07900	mitochondria	N.D. *^3^	
mTERF8 (PTAC15)	AT5G54180	chloroplasts	No obvious phenotype	[41]
mTERF9 (TWIRT1)	AT5G55580	chloroplasts	Pale green / Plastid gene expression	[38, 42]
mTERF10	AT2G34620	chloroplasts	No obvious phenotype / salt stress response?	[28, 43]
mTERF11	AT3G18870	chloroplasts	No obvious phenotype / salt stress response?	[28, 43]
mTERF12	AT4G09620	chloroplasts	No obvious phenotype	[28, 43]
mTERF13	AT1G61990	mitochondria	N.D.	
mTERF14	AT1G62010	mitochondria	N.D.	
mTERF15	AT1G74120	mitochondria	embryo arrest / group II introns splicing	[44]
mTERF16	AT1G78930	chloroplasts	embryo arrest / maturation of tRNA^Ile^ (GAU)	[28]
mTERF17	AT1G79220	mitochondria	N.D.	
mTERF18 (SHOT1)	AT3G60400	mitochondria	Increased heat tolerance	[45]
mTERF19	AT5G06810	N.D.	N.D.	
mTERF20	AT1G62150	mitochondria	N.D.	
mTERF21	AT2G44020	mitochondria	N.D.	
mTERF22	AT5G64950	mitochondria	No obvious phenotypes under optimal growth conditions / mild reductions in the levels of many mtRNAs	(This study)
mTERF23	AT1G56380	N.D.	N.D.	
mTERF24	AT1G62085	N.D.	N.D.	
mTERF25	AT1G62490	N.D.	N.D.	
mTERF26	AT4G19650	N.D.	N.D.	
mTERF27	AT1G21150	mitochondria	N.D.	
mTERF28	AT1G61960	mitochondria	N.D.	
mTERF29	AT1G61970	N.D.	N.D.	
mTERF30	AT1G61980	mitochondria	N.D.	
mTERF31	AT1G62110	mitochondria	N.D.	
mTERF32	AT1G62120	mitochondria	N.D.	
mTERF33	AT3G46950	mitochondria	N.D.	
mTERF34	AT5G23930	N.D.	N.D.	
mTERF35	AT5G45113	cytoplasm/nucleus	N.D.	

*^1^ ‒ Gene annotations according to Kleine 2012 [46]

*^2^ ‒ According to Babiychuk 2011 [28]

*^3^ ‒ N.D.–not determined

Loss-of-function mutants of several plastid-localized members of the mTERF family in plants show altered stress responses [43], defects in chloroplast gene expression [38], and altered plastidial RNA (cpRNA) metabolism [28, 33, 35, 39, 47]. Likewise, mutations in the mitochondrial *MOC1* gene (mTERF-like gene of *Chlamydomonas* 1) reduce the transcript levels of the complex I subunit *nad1* gene and affect the biogenesis of the respiratory machinery in the alga [48–50]. In Arabidopsis, mTERF15 was recently shown to be involved in the processing of *nad2* pre-RNA in the mitochondria [44]. It is, therefore, anticipated that other members of this family in plants would carry important roles in RNA metabolism in plant mitochondria. In this work we analyzed the roles of mTERF22, encoded by the At5g64950 gene-locus in Arabidopsis. The effects of lowering the expression of mTERF22 on the organellar functions and physiology of knockout mutant-lines in Arabidopsis are discussed.

## Materials and methods

### Plant material and growth conditions

Arabidopsis thaliana (ecotype Columbia) was used for the genetic analysis of mTERF22. Wild-type and individual mutant lines (i.e., T-DNA insertional lines) were obtained from the Arabidopsis Biological Resource Center (ABRC, Columbus, OH). Tobacco (*Nicotiana benthamiana*) was used for GFP localization studies. Prior to germination, seeds of wild-type and mutant lines were surface sterilized by a vapor-phase method, with 50 ml sodium hypochlorite (bleach, 6%) solution containing 1.5 ml HCL, and sown on MS-agar plates containing 1% (w/v) sucrose. The seeds were kept in the dark for 5 days at 4°C and then germinated in a controlled temperature and light conditions (i.e., 22°C or 28°C, light intensity of 150 μE·m^-2^·s^-1^) in a growth chamber (Percival Scientific, Perry, IA, USA). Soil-grown plants were cultivated in growth chambers, under similar growth conditions (i.e., 22°C or 28°C, 50% RH and light intensity of 150 μE·m^-2^·s^-1^) in either short (SD 8:16-hour) or long (LD 16:8-hour) day conditions. PCR was used to screen the plant collection. Specific oligonucleotides are listed in S1 Table. Sequencing of PCR products was used to analyze the precise insertion sites in each T-DNA line.

### Microscopic analyses

For the analysis of plant morphology plant organs where obtained from wild-type and homozygous lines, at specific developmental stages and examined under a Stereoscopic (dissecting) microscope. The morphologies of mitochondria (and plastids) were established by transmission electron microscopy (TEM) of ultrathin sections of young (4~7 day-old) wild-type (Col-0) and *mterf22* seedlings, grown on sucrose-containing MS medium plates, at the Bio-Imaging unit of the Institute of Life Sciences (The Hebrew University of Jerusalem).

### GFP localization assays

A 300 nucleotide fragment of the N-terminus of mTERF22 (At5g64950) was fused in-frame to eGFP, and cloned into pSAT6 vector [51]. The resulting construct (*N*-mTERF22-GFP) was cloned into the binary pCAMBIA-2300 vector (Specific primers are listed in S1 Table). The vector was introduced into *Agrobacterium tumefaciens* (strain EHA105) by electroporation (1.8 kV, 25 μF, and 200 Ω), using Gene pulser II (Bio-Rad). The transformed *A*. *tumefaciens* cells were grown overnight at 28°C in YEP medium supplemented with kanamycin (50 mg/L). The cells were harvested by centrifugation and resuspended to a final concentration equivalent to 1.0 O.D. at 600 nm in a solution containing 0.5 M MgCl_2_, 0.5 M MES pH 5.6 and 0.1 M acetosyringone. For the localization analyses, three young leaves of *N*. *benthamiana* (4 to 6 week-old) were infiltrated with recombinant Agrobacterium strains using a 1 mL syringe (abaxial side, without a needle). GFP alone and GFP fused to N terminal region of the mitochondrial AtpB subunit [52, 53] were used as a controls. After 24~48 hours, the Agro-infiltrated leaves were analyzed by confocal microscopy (Olympus IX 81, Fluoview 500, Imaging facility, Volcani center). To visualize mitochondria in vivo, plant tissue was incubated with 2 μM MitoTracker^®^ for 10 min at room temperature prior to the confocal analyses.

### Respiration activity

Oxygen consumption (O_2_-uptake) measurements were performed with a Clarke-type oxygen electrode (Oxygraph, Hansatech Instruments, Norfolk, UK), and the data feed was collected by Oxygraph-Plus software as described previously [54]. The electrode was calibrated with O_2_-saturated water, and by depletion of the oxygen in the electrode chamber with the addition of excess sodium dithionite. Equal weights of three week-old seedlings (about 100 mg fresh tissue) were immersed in autoclaved tap water and incubated in the dark for a period of 30 min. Total respiration was measured at 25°C in the dark following the addition of plants to 2.5 ml of water in the respiration chamber.

### Mitochondria preparation from MS-grown Arabidopsis seedlings

Isolation of mitochondria from Arabidopsis plants was performed as described previously [55]. About 10 gr MS-grown Arabidopsis seedlings were grinded with 500 ml extraction buffer (0.3 M Sucrose, 10 mM KH_2_PO_4_, 5 mM tetrasodiumpyrophosphate, 2 mM EDTA, 1 mM DTT, 5 mM Cysteine, 1% Polyvinylpyrrolidone molecular weight 40,000 (PVP-40), 1% BSA, pH 7.5). The plant lysate was filtrated through two layers of miracloth and centrifuged at 2,500 ×g for 4 min (to remove cell debris) and then at 20,000 ×g for 20 min. The resultant pellet, containing intact chloroplasts and mitochondria was resuspended in a small volume (2~3 ml) of wash buffer (0.3 M sucrose, 10 mM MOPS, 1 mM EGTA, pH 7.2), homogenized and loaded onto a 18%-25%-50% Percoll step gradient. The mitochondria fraction was obtained by ultracentrifugation at 40,000 xg for 45 min (mitochondria form a yellowish band between the 25 and 50% layers of the Percoll gradient). For immunoassays, mitochondria were suspended in sample loading buffer [56] and subjected to SDS-PAGE (at a constant 100 V). Following electrophoresis, the proteins were transferred to a PVDF membrane (BioRad) and incubated overnight (at 4°C) with various primary antibodies (S2 Table). Detection was carried out by chemiluminescence assay after incubation with HRP-conjugated secondary antibody.

### Organellar protein extraction and analysis

Arabidopsis organellar proteins were prepared essentially as described previously [57]. Crude mitochondria membrane extracts were obtained from 200 mg MS-grown Arabidopsis seedlings. The plant tissue was homogenized in 2 ml of 75 mM MOPS-KOH, pH 7.6, 0.6 M sucrose, 4 mM EDTA, 0.2% polyvinylpyrrolidone-40, 8 mM cysteine, 0.2% bovine serum albumin (BSA) and protease inhibitor cocktail ‘complete Mini’ from Roche Diagnostics GmbH (Mannheim, Germany). The lysate was filtrated through one layer of miracloth and centrifuged at 1,300 ×g for 4 min (to remove cell debris). The supernatant was then centrifuged at 22,000 ×g for 20 min. The resultant pellet enriched with mitochondrial membranes was washed with 1 ml of wash buffer 37.5 mM MOPS-KOH, 0.3 M sucrose and 2mM EDTA, pH 7.6. Protein concentration was determined by the Bradford method (BioRad, Catalog no. 5000201) according to the manufacturer's protocol, with bovine serum albumin (BSA) used as a calibrator. For immunoassays, crude mitochondria fractions were suspended in sample loading buffer [56] and subjected to SDS-PAGE (at a constant 100 V). Following electrophoresis, the proteins were transferred to a PVDF membrane (BioRad, Catalog no. 1620177) and incubated overnight (at 4°C) with various primary antibodies (S2 Table). Detection was carried out by chemiluminescence assay after incubation with an appropriate horseradish peroxidase (HRP)-conjugated secondary antibody.

### Blue native (BN) gel electrophoresis for isolation of native organellar complexes

Blue native (BN)-PAGE of mitochondria preparations was performed generally according to the method described by Pineau 2008 [57]. Mitochondria obtained from wild-type and mterf22 plants were solubilized with n-dodecyl-ß-maltoside (DDM; 1.5% [w/v]) in ACA buffer (750 mM amino-caproic acid, 0.5 mM EDTA, and 50 mM Tris-HCl, pH 7.0), and then incubated on ice for 30 min. The samples were centrifuged 8 min at 22,000 xg to pellet any insoluble materials. Serva Blue G was added to the clear supernatant to a final concentration of 0.2% (v/v). The samples were then loaded onto a native 4 to 16% linear gradient gel. For immunoblotting of non-denaturing PAGE, the proteins were transferred from the gel onto a PVDF membrane (Bio-Rad) in Cathode buffer (50 mM Tricine, 15 mM Bis-Tris-HCl, pH 7.0) for 16 h at 4°C at constant current of 40 mA. The membranes were then incubated with specific primary antibodies (S2 Table), and detection was carried out by chemiluminescence assay after incubation with horseradish peroxidase (HRP)-conjugated ‘secondary’ antibodies.

### RNA extraction and analysis

Total RNA was extracted from Arabidopsis seedlings by TRIzol (Termo Fisher Scientific), according to the manufacturer's instructions. The RNA was then treated with DNase I (RNase-free) (Ambion, Thermo Fisher Scientific) to remove DNA contamination. RT-qPCR was performed with specific oligonucleotides (S3 Table), essentially as described previously [58–61]. Reverse transcription was carried out with the Superscript III reverse transcriptase (Invitrogen), using 1–2 μg of total RNA and 250 ng of a mixture of random hexanucleotides (Promega) and incubated for 50 min at 50°C. The RT reactions were stopped by 15 min incubation at 70°C and the RT samples served directly for real-time PCR. Quantitative PCR (qPCR) reactions were run on a 384-well LightCycler 480^®^ Instrument II (Roche, Product no. 05015243001), using 2.5 μl of LightCycler 480 SYBR Green I Master mix and 2.5 μM forward and reverse primers in a final volume of 5 μl. Reactions were performed in triplicate in the following conditions: pre-heating at 95°C for 10 min, followed by 40 cycles of 10 sec at 95°C, 10 sec at 58°C and 10 sec at 72°C. The nucleus-encoded 18S rRNA (At3g41768) and *actin2* (At3g18780) were used as reference genes. The specific primers for each gene in *Arabidopsis thaliana* (Col-0) were designed according to the guidelines by LightCycler^®^ 480 SYBR Green I Master (Roche, Product no. 04887352001) for appropriate melting temperature of 58.0°C and PCRs product lengths between 90 bp to 120 bp (S3 Table and Ref’s [59–63]).

### Mitochondrial ‘run-on’ transcription assays

Run-on transcription assays were preformed as described previously [64]. Freshly prepared mitochondria, obtained from 3-week-old MS-plates grown wild-type and *mterf22* plants, were used in a nonradioactive labeling of organellar transcripts for the subsequent detection of ‘run-on’ mtRNAs. For this purpose, mitochondria were pelleted (10 min at 20,000 xg) and resuspended in a run-on mix (20 mM Tris pH 7.6, 40 mM KCl, 10 mM MgCl 2, 1 mM DTT, 0.5 mM of each ATP, GTP and UTP, 0.3 mM CTP, 1 mM biotin-CTP, 40 U RNase inhibitor). Following 1 hour incubation, total mtRNA was obtained by phenol/chloroform extraction and ethanol precipitation. Northern blotting [58] was used for the analyses of de-novo transcription. mtRNA preparations following the run-on transcription were separated on agarose gel electrophoresis and transferred to a charged nylon membrane. Transcriptional rates analyses were preformed according to the method described in [64]. Following membrane hybridization and washes, detection was carried out by chemiluminescence assay after incubation with streptavidin-HRP conjugated antibodies.

## Results

### The At5g64950 gene-locus encodes an mTERF-related protein (mTERF22) that is localized to the mitochondria in vivo

**Fig 1:**
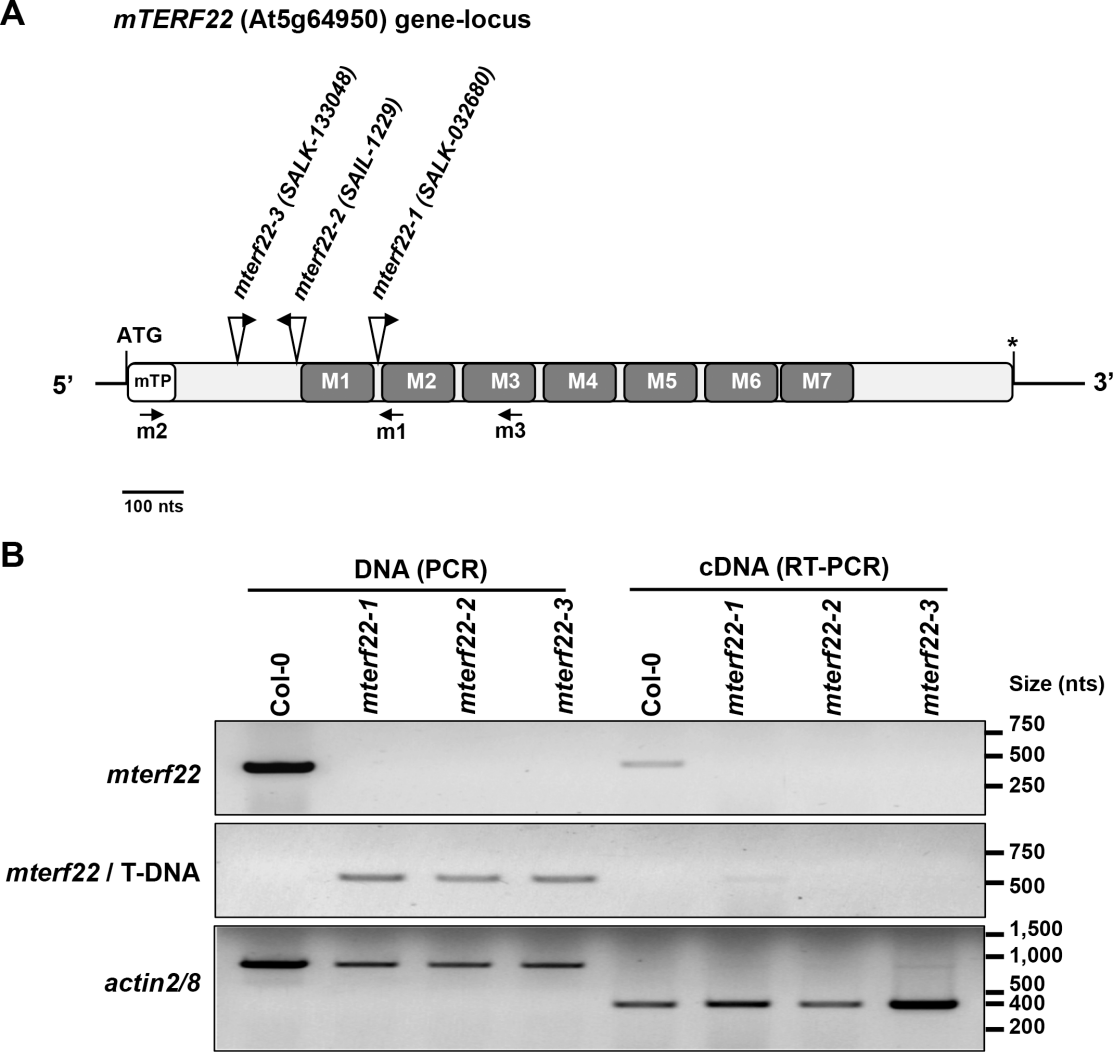
*Arabidopsis thaliana* (Col-0) mTERF22 structure. (A) Schematic representation of mTERF22 (At5g64950). The initiation (ATG) site and the stop codon (marked by asterisk) are outlined in the figure. The deduced N-terminal organelle targeting signal (mTP), and the seven MTERF domain are highlighted within the sequence, according to the Conserved Domain Database (CDD) [68] and SMART [69] servers. The locations of the T-DNA insertion sites in *mterf22-1* (SALK- 032680), *mterf22-2* (SAIL-1229) and *mterf22-3* (SALK-133048) are indicated above the sequence (see also S2 Fig). Arrows indicate the positions of the left borders (LBs) in the T-DNA insertional lines. (B) Mutant genotypes were confirmed by PCR with oligonucleotides designed to gene-specific and T-DNA sequences (LBs; about 500 nts upstream or downstream of the T-DNA insertion) (i.e., pROK2 and pCSA110 vectors for SALK and SAIL lines, respectively), followed by sequencing of the ~500 nts PCR products. Specific oligonucleotides are shown below mTERF22 gene scheme (the oligonucleotides m1 for the screening of *mterf22-1*, m2 for *mterf22-2*, and m3 for *mterf22-3* mutants) and their nucleotides sequences are listed in S1 Table. The relative accumulation of mRNA transcripts corresponding to mTERF22 in wild-type plants (Col-0) and *mterf22* mutants was analyzed by RT-PCRs with gene-specific oligos (S1 Table). ACTIN2/8 (At3g18780) was used as a control in the reaction. The bar in panel ‘A’ represents 100 nucleotides.

Proteins which contain the MTERF domain were shown to function in various aspects of organellar (i.e., mitochondria or chloroplasts) gene expression and RNA metabolism, in both animals and plants. Arabidopsis encodes at least 35 mTERFs, many of which harbor N-terminal extension regions with sequence similarities to mitochondria and/or plastid targeting signals [28, 30, 31]. A list of the 35 mTERF members in *Arabidopsis thaliana*, their functions and intracellular loci can be found in Table 1. Here, we examined the roles of mTERF22, encoded by the At5g64950 gene-locus (Fig 1), considering the importance of mTERFs to organellar gene expression and RNA metabolism in ‘higher’ eukaryotes. Analysis of the expression profiles of the At5g64950 locus, available in the ‘Genevestigator microarray database’ [65] and AtGeExpress [66], suggest that mTERF22 is expressed at low levels in different tissues throughout the plant development (S1 Fig). mTERF22 is postulated to resides within the mitochondria [28], but no peptides corresponding to the mTERF22 protein could be identified in mass-spectrometry analyses of Arabidopsis organellar proteomes (SUBA3 server, [67]). However, this might be expected in light of the difficulties in detecting proteins expressed at a low level.

**Fig 2:**
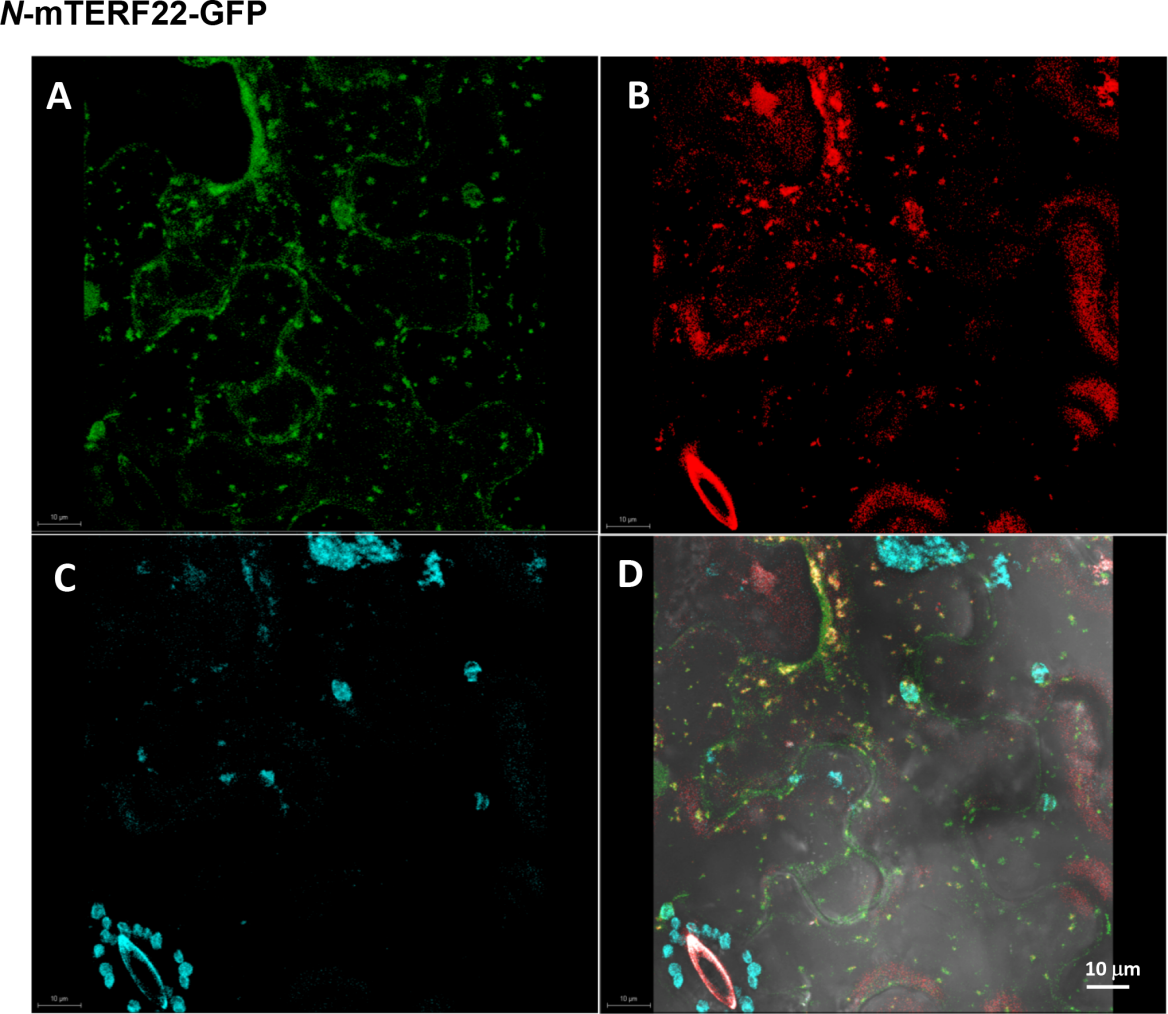
GFP localization analysis indicates that mTERF22 protein resides within the mitochondria. Tobacco leaves were agroinfiltrated with GFP fused to the N-terminal region (100 amino acids) of the Arabidopsis mTERF22 (At5g64950) protein (see ‘Materials and Methods). GFP alone and GFP fused to the N-terminal region (54 amino acids) of ATP synthase b-subunit were used as controls for the integrity of the GFP localizations assays (see ‘Materials and Methods and S3 Fig). The GFP signals (green, upper left), MitoTracker marker (red, upper right), chlorophyll autofluorescence (blue, lower left) and merged images (lower right), are outlined in each panel.

To establish the intracellular location(s) of mTERF22 protein, a construct encoding the N-terminal region of *mTERF22* gene was cloned in-frame to GFP, introduced into tobacco leaves by Agroinfiltration, and the intercellular location of the fusion mTERF22-GFP protein was examined by confocal microscopy (see Materials and Methods). RACE analyses were used to ensure the integrity of the start codon of mTERF22 in Arabidopsis (S2 Fig). As controls, we used GFP alone and a construct expressing the N-terminal region (54 amino acids) of the mitochondria localized ATP-synthase subunit B (AtpB) protein fused in frame to GFP [52, 53]. GFP alone was observed in the cytoplasm, whereas the signal of AtpB-GFP was detected as rod-shaped granules, co-localizing with those of mitochondrial MitoTracker marker (S3 Fig). Fluorescence signals within the nucleus that are apparent in the cases of both GFP and AtpB-GFP likely correspond to non-specific diffusions of these relatively small proteins through the nuclear pores [70]. Next, we analyzed the intracellular location of mTERF22 protein, in vivo. In agreement with published data [28] and its predicted localization to the mitochondria, the signal of the GFP-mTERF22 protein co-localized with the MitoTracker labeled mitochondria (Fig 2), further indicating that the At5g64950 encodes a mitochondria-localized protein.

### The topology of mTERF22 protein shares structural similarities with mammalian mTERFs

mTERFs proteins are characterized by the repetition of about 30 amino acids region, termed as MTERF motif [27]. The presence and topology of MTERF and other conserved motifs in mTERF22 were analyzed and identified using the Web-based tools Conserved Domains Database (CDD) (https://www.ncbi.nlm.nih.gov/Structure/cdd/cdd.shtml, [68]) and Simple Modular Architecture Research Tool (SMART) (http://smart.embl-heidelberg.de, [69]). Alignment of mTERF7 was conducted with T-Coffee multiple sequence alignment server [71] and displayed using GeneDoc [72] with the conserved residue shading mode (see S1 Fig). According to the CDD and SMART servers, the sequence of mTERF22 protein contains seven MTERF motifs (i.e., amino acid regions 89–125, 125–156, 162–194, 199–229, 233–263, 268–298, 300–334), which are connected by short linker regions (Figs 1A and S2). To gain structural insights, we used the Protein homology/analogy recognition engine-2 (Phyre^2^) [73] to predict and generate models of the mTERF22 structure. Based on the Phyre^2^ server, mTERF22 adopts a structure that shares similarity with the established folds of the mTERF1 and mTERF3 proteins of animals (S4 Fig; and [74–77]). The hypothetical 3D-structure of mTERF22 has as a solenoid fold, with various exposed basic surfaces that are postulated to function in nucleic-acid binding (S4 Fig). A similar architecture is also recognized in various transcription factors in eukaryotes [78].

### Morphology of mTERF22 mutant-alleles

The Arabidopsis genome encodes 35 mTERF proteins, many of which are predicted to reside within the mitochondria or chloroplasts (see Table 1). These are expected to play important roles in organellar gene expression in plants, and hence to affect the biogenesis of the respiratory and/or photosynthetic machineries. To investigate the roles of mTERF22, we examined several T-DNA insertional lines available in The Arabidopsis Information Resource (TAIR), which are affected in the At5g64950 gene-locus (i.e., SALK-032680, SAIL-1229 and SALK-133048, annotated here as *mterf22-1*, *mterf22-2* and *mterf22-3*, respectively). PCR analysis and DNA sequencing confirmed the positions of the insertions within the coding region of mTERF22 in *mterf22-1*, *mterf22-2* and *mterf22-3* mutants (Figs 1A and S2).

**Fig 3:**
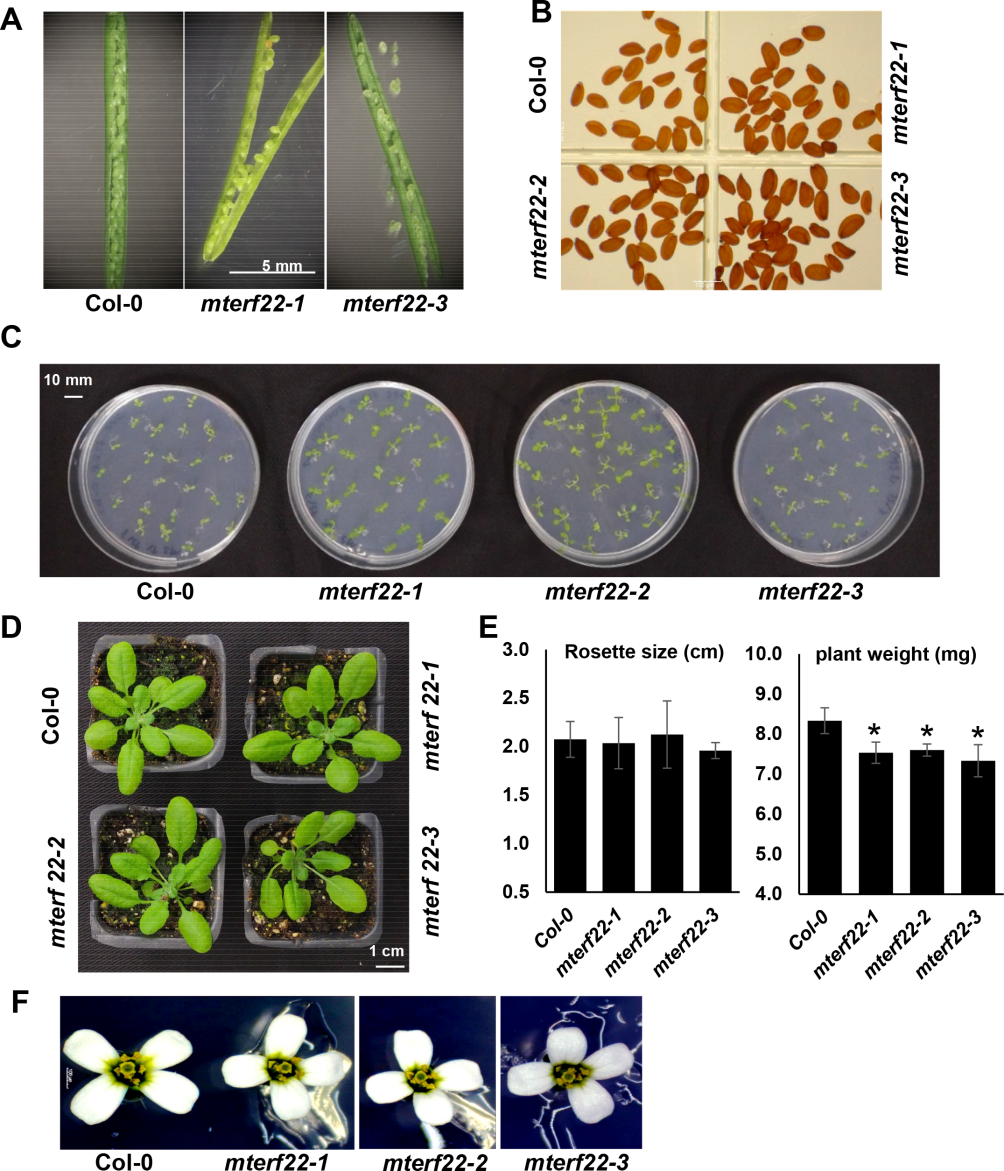
Plant phenotypes associated with mTERF22 mutations. The effects of *mTERF22*-suppression on germination, growth phenotypes and development of Arabidopsis wild-type (Col-0) and *mterf22* knockout lines (i.e., SALK-032680, SAIL-1229 and SALK-133048, annotated here as *mterf22-1*, *mterf22-2* and *mterf22-3* mutants, respectively). The morphologies of siliques (A) and seeds (B) obtained from wild-type (Col-0) and *mterf22* mutant lines analyzed under a microscope. (C) Growth phenotypes associated with 3-week-old wild-type and homozygous *mterf22* seedlings grown on MS-agar plates at 22°C. (D) Growth phenotypes associated with 7-week-old wild-type and *mterf22* seedlings grown on soil at 22°C. (E) The averages of seedlings weights and rosette sizes of 3 week-old and 7 week-old (respectively) wild-type plants and *mterf22* mutants. The values are means of three biological replicates with about 20 ~ 25 seedlings from each line in each batch. Error bars indicate one standard deviation. Asterisks indicate significant differences between the wild-type and *mterf22* mutant-lines, using Student’s paired t-test (P < 0.05). (F) Flower phenotypes of *mterf22* mutants grown at 22°C under long-day conditions (i.e., 16:8-hour) at light intensity of ~150 μE m^-2^ s^-1^, with 50% relative humidity.

A downregulation in the expression of factors required in mitochondrial gene expression in Arabidopsis may result with growth and developmental retardation phenotypes, and in some cases were shown to lead to the production of abnormal seed morphology (reviewed by e.g., [79]). Our characterization of Arabidopsis mutants affected in the expression of nuclear genes homologous to intron-encoded splicing cofactors in bacteria (i.e., nuclear-encoded maturases, or *nmat*), indicated that they affect the splicing of various mitochondrial genes [52, 60, 63, 80]. The seed morphology of *nmat1*, *nmat2 and nmat4* mutants [52, 60, 63, 80, 81] resembled that of the *wrinkled* mutant [82]. Accordingly, the three *nmat* also exhibited lower germination and retarded growth phenotypes [52, 60, 63, 80]. Yet, morphologically, the homozygous *mterf22-1*, *mterf22-2*, *mterf22-3* mutants were indistinguishable from the wild-type plants when they are grown under ‘standard’ growth conditions (i.e., 22°C, 16:8-hour long-day conditions, 150 μE m^-2^ s^-1^, 50% relative humidity) (Fig 3). Analyses of fully developed siliques from heterozygous lines (Fig 3A) and mature seeds obtained from homozygous *mterf22* plant lines (Fig 3B) indicated the presence of only normal seeds in *mterf22* mutants. Likewise, no obvious changes in germination rates (S4 Table), rosette size (Fig 3D and 3E) and flower morphology (Fig 3F) were seen between the mutants and wild-type plants, though the average weights of the *mterf22* seedlings may be somewhat lower (about 10%) than that of the wild-type plants (Fig 3E). However, when are grown at 28°C (i.e., 16:8-hour long-day conditions, 150 μE m^-2^ s^-1^, 70% relative humidity) the *mterf22* mutants show reduced germination rates (S4 Table) and altered root morphology (S5 Fig).

### Homozygous *mterf22* mutants exhibit mitochondrial biogenesis defects

**Fig 4:**
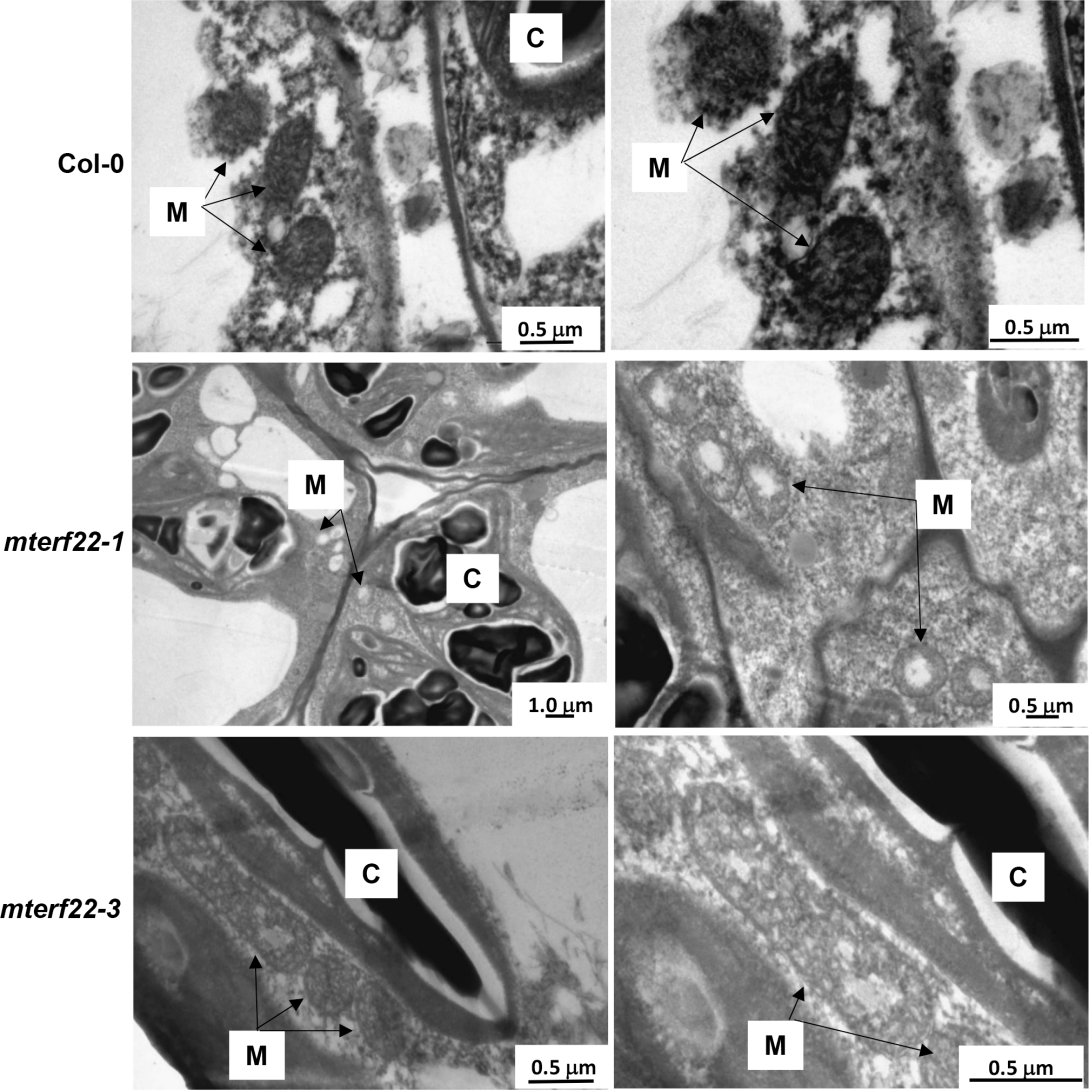
Mitochondria morphologies of Arabidopsis wild-type and *mterf22* plants. Transmission electron micrographs of ultrathin sections from the hypocotyl tissue of wild-type and *mterf22-1* and *mterf22-3* mutants. The right panels are magnified images of the micrographs presented in the left panel. Mitochondria are labeled by ‘M’, while ‘C’ indicates to chloroplasts. Bars represent 0.5 μm or 1.0 μm as indicated in each panel.

To evaluate the impact of elevated ambient temperature conditions on mitochondria biogenesis and organellar gene expression, wild-type and *mterf22* plants were germinated at 22°C and then transferred for four or five days at 28°C (unless indicated differently). The biogenesis of wild-type and *mterf22* mitochondria was analyzed by transmission electron microscopy (TEM) in ultrathin sections obtained from young Arabidopsis seedlings (i.e., germinated at 22°C and then grown for 5 days at 28°C). The electron micrographs of wild-type mitochondria showed the characteristic internal cristae formation [52, 59, 60, 63], as dense folds of the inner-membrane sections (Fig 4). Many of the mitochondria in *mterf22* mutants were less electron densed, and showed reduced inner mitochondrial membrane (IMM) electron density, with less cristae organization and often had large internal spaces lacking any obvious IMM formation (Fig 4). One of the mitochondria in *mterf22-3* was particularly elongated (Fig 4, lower panel). As this organellar phenotype was observed only in a single slide, we assume that the morphology of the elongated mitochondria in *mterf22-3* corresponds to a fusion between two neighboring mitochondria, a phenomenon that is common to land-plants [83]. As Arabidopsis mutants affected in mitochondria gene expression also exhibit similar organellar morphologies (i.e., less developed cristae formation (see e.g., [52, 59, 60, 63]), we assume that the functions of mTERF22 are required for normal mitochondrial biogenesis.

### Depletion of mTERF22 leads to decreased transcript levels of many mitochondrial genes

**Fig 5:**
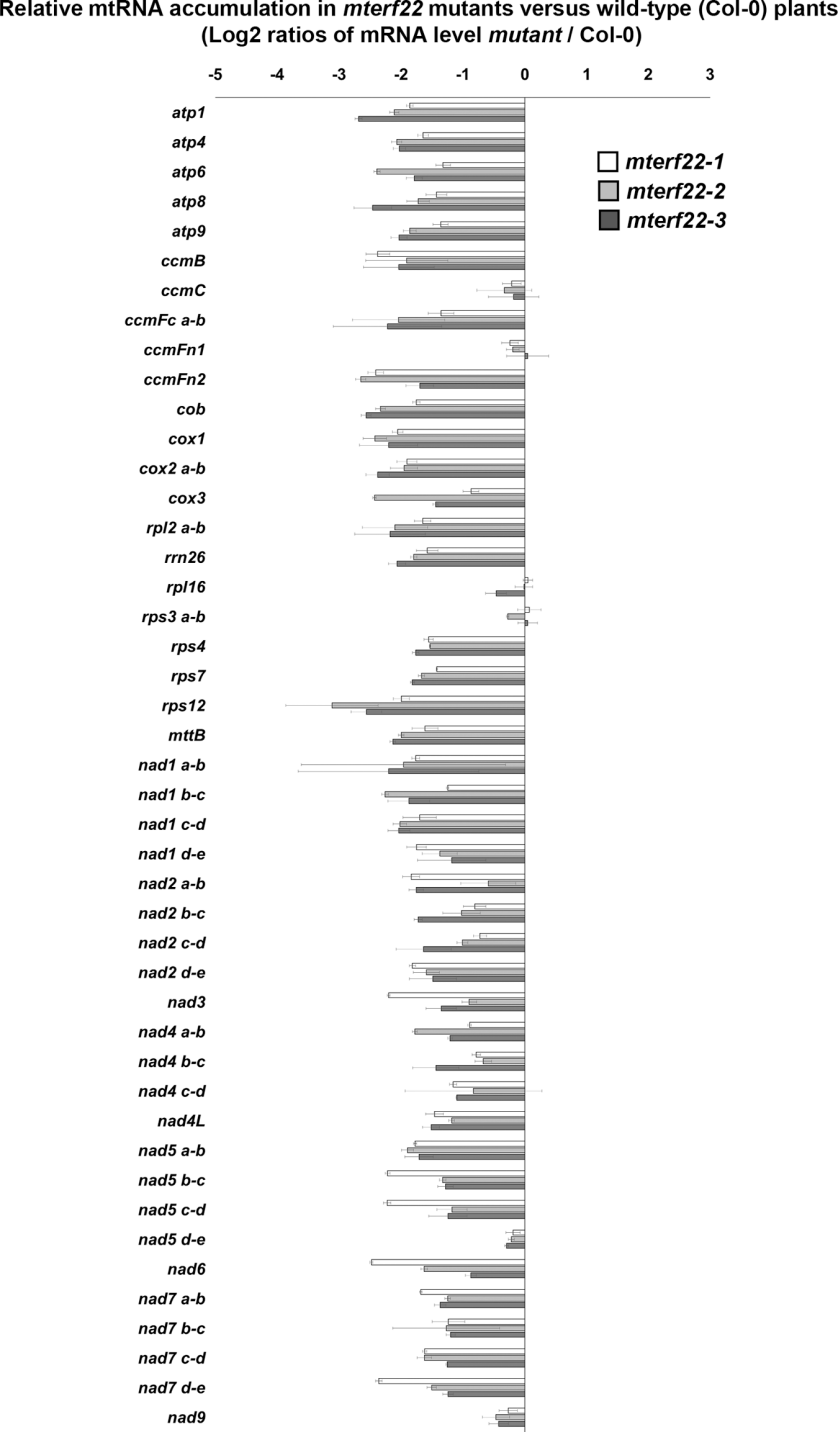
Transcript abundance of mitochondrial mRNAs in Arabidopsis wild-type and *mterf22* plants grown at 28°C. The accumulation of various mtRNAs in wild-type and *mterf22* plants grown at 28°C was analyzed by quantitative reverse transcription PCR (RT-qPCR). Total RNA (depleted from DNA) was extracted from 3-week-old wild-type (Col-0) and mutant plants, reverse-transcribed, and the relative steady-state levels of different organellar transcripts were evaluated by qPCR with specific oligonucleotides, after normalization to the *actin2* (At3g1878), 18S rRNA (At3g41768) and 26S rRNA (Atmg00020) genes (see S3 Table). The histogram shows the relative mRNAs levels in mutant lines versus those of wild-type plants (i.e., log2 ratios of mutant to wild-type mtRNAs). Transcripts analyzed in these assays include the NADH dehydrogenase (i.e., complex I) subunits *nad1* exons a-b, b-c, c-d, d-e, *nad2* exons a-b, b-c, c-d, d-e, *nad3*, *nad4* exons a-b, b-c, c-d, *nad4L*, *nad5* exons a-b, b-c, c-d, d-e, the intronless *nad6* subunit, *nad7* exons a-b, b-c, c-d, d-e, and *nad9*, the complex III *cob* subunit, cytochrome oxidase (complex IV) *cox1*, *cox2* exons a-b and *cox3* subunits, the ATP synthase (i.e., complex V) subunits *atp1*, *atp4*, *atp6*, *atp8* and *atp9*, genes encoding the cytochrome c maturation (*ccm*) factors, *ccmB*, *ccmC*, *ccmFn1*, *ccmFn2* and *ccmFc* exons a-b, the ribosomal subunits *rpl2* exons a-b, *rps3* exons a-b, *rps4*, *rps7*, *rps12*, *rpl16*, *rrn26*, and the *mttB* gene. The values are means of five biological replicates (error bars indicate one standard deviation).

The abnormal mitochondrial morphology and growth phenotypes associated with *mterf22* mutants suggest that mTERF22 may functions in mitochondrial RNA metabolism. To address specific changes in the accumulation of various mitochondrial transcripts in *mterf22* plants, we compared the RNA profiles of wild-type plants and *mterf22* mutants. For this purpose, total RNA was extracted from 3-week-old Col-0, *mterf22-1*, *mterf22-2* and *mterf22-3* plants and the relative accumulation of different mitochondrial transcript in wild-type and *mterf22* plants was examined by RT-qPCR analyses with specific oligonucleotides designed to different organellar transcripts (see e.g., [59–63]). Genes analyzed in these assays included most of the annotated genes in Arabidopsis mitochondria [84]. These include the complex I subunits *nad1* exons a-b, b-c, c-d, d-e, *nad2* exons a-b, b-c, c-d, d-e, *nad3*, *nad4* exons a-b, b-c, c-d, *nad4L*, *nad5* exons a-b, b-c, c-d, d-e, *nad6*, *nad7* exons a-b, b-c, c-d, d-e, the *nad9* subunit, the complex III *cob* subunit, the complex IV *cox1*, *cox2* exons a-b and *cox3* subunits, the ATP synthase (i.e., complex V) subunits *atp1*, *atp4*, *atp6*, *atp8* and *atp9*, genes encoding the cytochrome c maturation (*ccm*) factors, *ccmB*, *ccmC*, *ccmFn1*, *ccmFn2* and *ccmFc* exons a-b, the ribosomal subunits *rpl2* exons a-b, *rps3* exons a-b, *rps4*, *rps7*, *rps12*, *rpl16*, *rrn26*, and the *mttB* gene. The ratios of transcripts accumulation between wild-type plants and *mterf22-1*, *mterf22-2* and *mterf22-3* mutants were examined for each transcript and are indicated in Fig 5. Specific oligonucleotides are listed in S3 Table.

Comparison of the RNA-profiles of wild-type and *mterf22* plants by RT-qPCR indicated discrepancies in the steady-state levels of various mitochondrial transcripts in *mterf22* mutants. Remarkably, these assays revealed to mild reductions (i.e., about x2 to x8 folds) in the accumulation of numerous mitochondrial transcripts in *mterf22-1*, *mterf22-2* and *mterf22-3* plants (Fig 5), strongly supporting a role for mTERF22 in the transcription, processing and/or stability of mitochondrial RNAs in Arabidopsis plants. Transcripts which their levels were reduced in the three mterf22 mutant lines corresponded to many genes which are interrupted by group II intron sequences in Arabidopsis mitochondria [85–87] (e.g., *ccmFc*, *cox2*, *nad1*, *nad2*, *nad4*, *nad5*, *nad7* and *rpl2*), as well as to numerous other intronless organellar transcripts. Yet, the accumulation of mtRNAs related to *ccmC*, *ccmFn1*, *rpl16*, *rps3* exons a-b, *nad5* exons d-e and *nad9*, was not significantly affected in *mterf22* (Fig 5).

Previously is was shown that at least two mTERF members function in the splicing of organellar-encoded group II introns in Arabidopsis plants (i.e., mTERF4 [35–37] and mTERF15 [44]). To test whether mTERF22 may also affect the maturation of various mitochondrial pre-mRNAs, we analyzed the splicing efficiencies of the 23 mitochondrial introns in Arabidopsis wild-type and *mterf22* mutant plants [84]. The ratio of unspliced and mature transcripts in wild-type and mutant plants was examined for each transcript. Splicing defects were concluded in cases where the accumulation of a pre-mRNA was correlated with a notable reduction in the level of the corresponding mRNA transcript in both mutants. However, we assume that the functions of mTERF22 are not required for splicing as no significant changes in the ratios of pre-RNAs to mRNAs of the 23 group II introns were observed between *mterf22* and wild-type plants (see S6 Fig).

Reduced transcripts levels in many mitochondrial genes in *mterf22* may also relate to altered mtDNA copy numbers, as previously indicated for some mTERFs in animals [88, 89] or algae [49]. Quantification of mtDNA copy number by qPCR analyses indicated that numbers of mtDNAs was not significantly affected in *mterf22-1* plants (S7 Fig). Taken together, these data indicate that mTREF22 affects the transcription or stability of many mitochondrial transcripts in Arabidopsis.

### Arabidopsis *mterf22* mutants are affected in mitochondrial transcription

**Fig 6:**
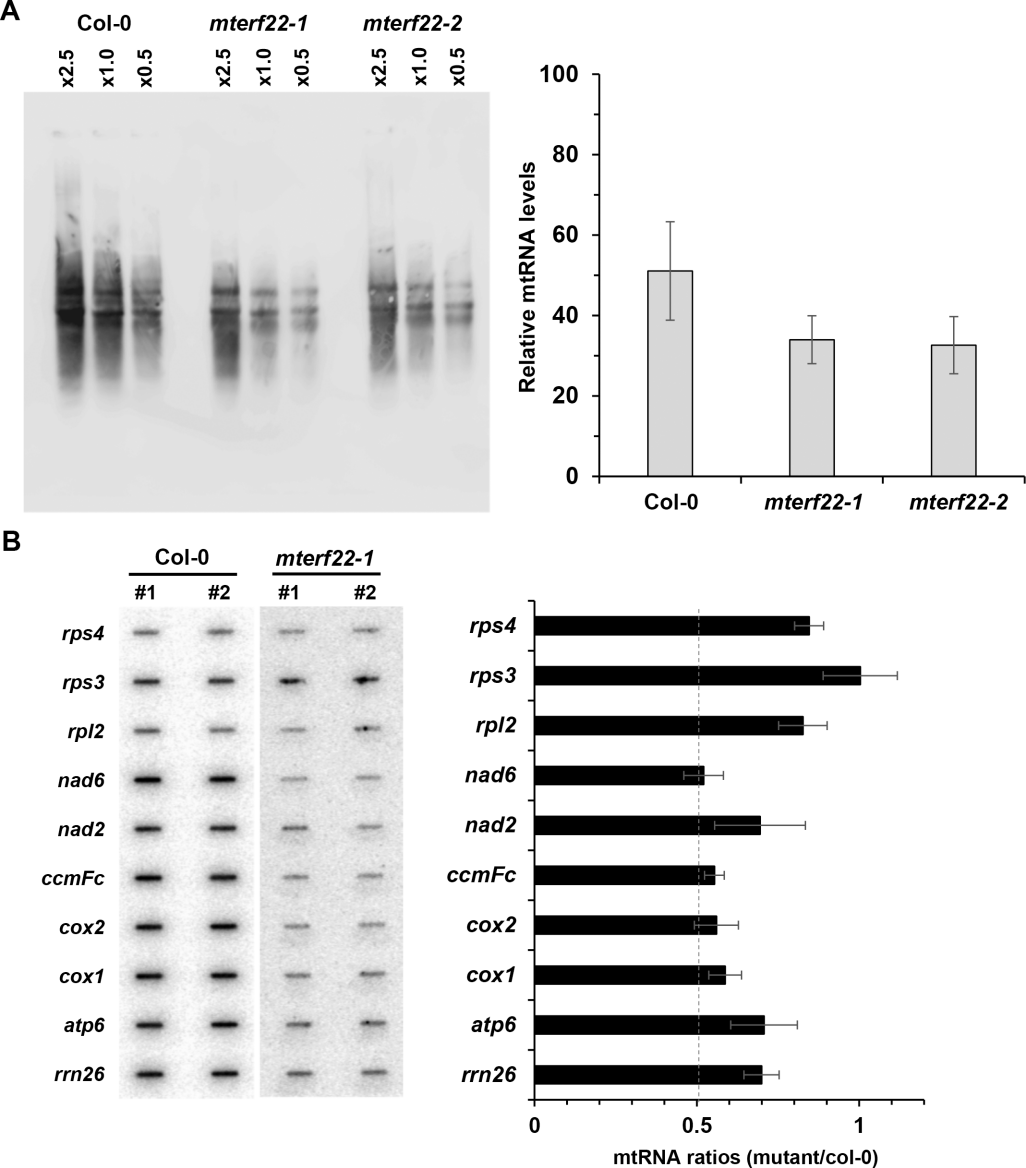
Mitochondrial run-on transcription assays. Mitochondria were prepared from 3-week-old MS-grown wild-type and *mterf22* plants, and run-on reaction was performed by incubation with ATP, GTP, UTP and biotin-14-CTP [64]. Following 1 hour incubation, total biotinylated-mtRNA was obtained by phenol/chloroform extraction and ethanol precipitation. (A) For the analyses of mtRNA transcription, total RNA (0.5, 1 and 2.5 μg) was separated on the 1% agarose gel and transferred to a charged nylon membrane, as previously described (Keren *et al*. 2011). The accumulation of various mitochondrial transcripts was estimated by measuring the signals in wild-type versus mutant plants, using the ImageQuant software (Molecular Dynamics, version 5.1). (B) Analysis of the transcription rates of specific mitochondrial genes was performed by the hybridization of the biotinylated-mtRNAs, extracted from wild-type and *mterf22-1* plants, with specific mtDNA gene probes (about 150 nts in length; S3 Table) immobilized onto charged nylon membranes, according to the method described by Kühn 2015 [64]. Following membrane hybridization and washes, detection was carried out by chemiluminescence assay after incubation with streptavidin horseradish peroxidase conjugated antibodies. The steady-state levels of various mitochondrial transcripts, as indicated in each blot, were analyzed the relative signals (i.e., mutant to wild-type ratios) in each blot, using the ImageQuant software (Molecular Dynamics, version 5.1). The values are means of two biological replicates (error bars indicate one standard deviation).

RNA synthesis and post-transcriptional processes shape the transcriptomic landscapes of plant mitochondria [9, 16, 64]. To establish whether mTERF22 functions in the transcription of mitochondrial genes or affects their stabilities, the transcriptional activities in *mterf22* were examined by run-on assays [64], using fresh mitochondria preparation obtained from wild-type and mutants plants. Intact mitochondria were incubated for 1 hour in the presence of a nucleotide mixture which consists of Biotin-14-CTP. Following the *in organello* run-on transcription assays, total mtRNA was isolated, separated on 1% agarose-gels and subjected to northern blotting with anti-biotin antibody. Relative mtRNA levels were measured by densitometry of RNA blots, and quantified using ImageJ software [90]. The results of the organellar run-on transcription assays indicated reduced de-novo transcription (*~*30% lower) in *mterf22* mutants mitochondria (Fig 6A). For the analysis of the transcription rates of specific mtRNAs, gene-specific probes (about 150 bp; see S3 Table) were blotted onto a positively charged nylon membrane, fixed by UV crosslinking and then hybridized with total mtRNAs obtained by the run-on assays. The relative accumulation of transcripts in wild-type plants and *mterf22* mutants was measured by the ImageQuant software (Molecular Dynamics, version 5.1). The levels of *de-novo* transcribed RNAs corresponding to *atp6*, *cox1*, *cox2*, *ccmFc*, *nad2*, *nad6* and *rrn26* were about 30% to 40% lower than those seen in the wild-type plants. Less marked reductions in mtRNA levels were seen in the cases of *rpl2* and *rps4* (~15% lower), while the transcription rates of *rps3* was not significantly affected in the mutant (Fig 6B). As significant amounts of mtRNAs are still observed in the mutants we conclude that mTERF22 is involved but is not essential for organellar transcription.

The RNA profiles and run-on experiments may indicate to functional redundancy, where various transcription factors are compensating for one another (see e.g., [91]). Based on phylogenetic analyses of the mTERF family in plants (S8A Fig and [28, 29, 46]), mTERF7 (At5g07900) and mTERF27 (At1g21150) are closely related to mTERF22. These also share a significant sequence homology with mTERF22 protein (i.e., 22% and 24% identity, respectively; S8B Fig) and are both predicted to reside within the mitochondria in plants (Table 1). It seems possible, therefore, that mTERF7 and mTERF27 (as well as other transcription factors) may have redundant functions with mTERF22 in the transcription of mitochondrial genes in Arabidopsis. However, such speculations should be considered with care. Genetic and biochemical data are required to clarify the roles of additional mTERF members in mtRNA metabolism in flowering-plants.

### *mterf22* mutants show mild reductions in the accumulation of different respiratory complexes

**Fig 7:**
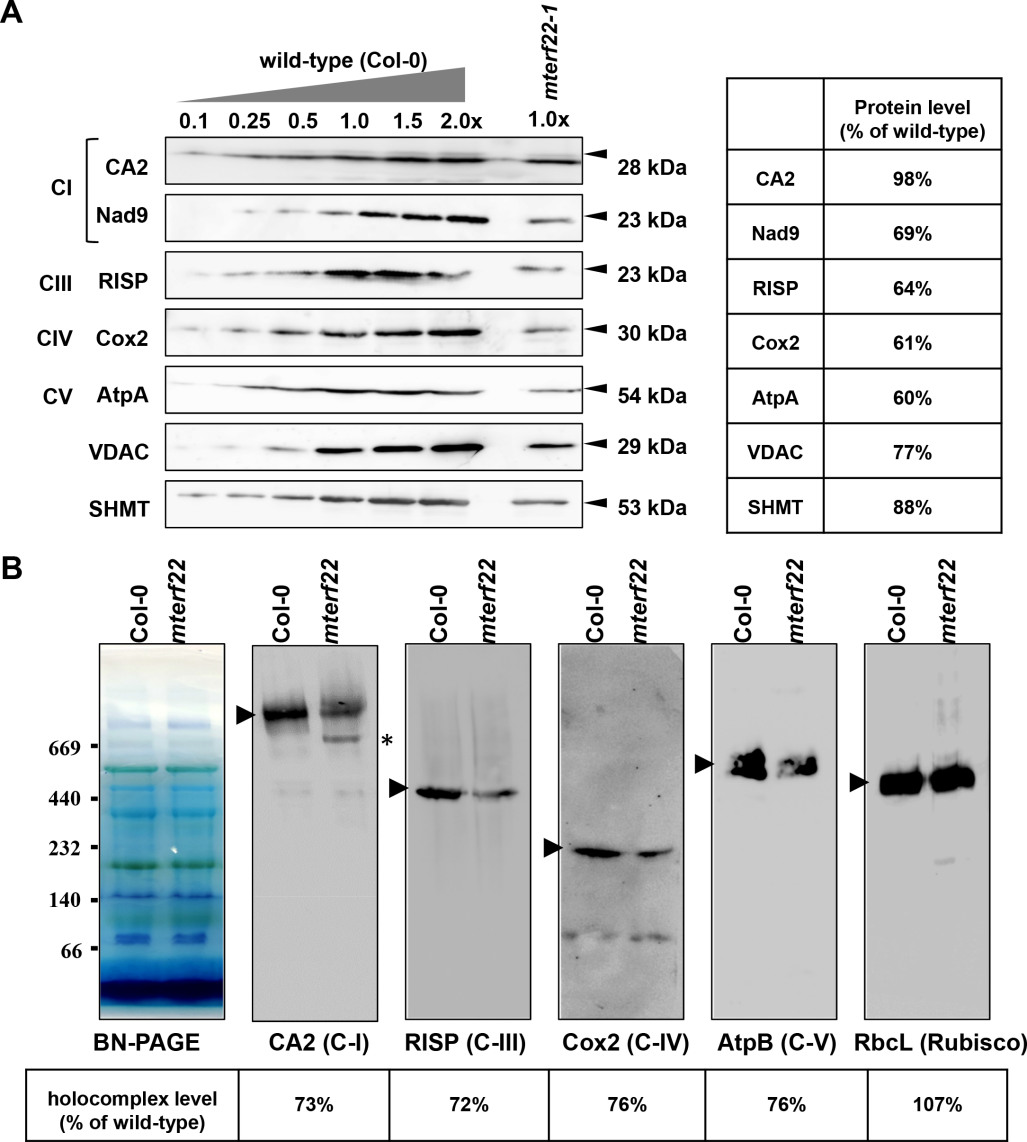
Relative accumulation of organellar proteins in wild-type and *mterf22* plants. (A) Immunoblot analyses of wild-type plants and *mterf22-1* mutant line. For the quantification of the relative abundances of organellar proteins in *mterf22* plants, different amounts of total mitochondrial proteins extracted from wild-type plants were loaded and separated by SDS-PAGE. The blots were probed with polyclonal antibodies raised to different organellar proteins, as indicated in each panel. Detection was carried out by chemiluminescence assays after incubation with HRP-conjugated secondary antibody. (B) BN-PAGE of crude mitochondria preparations was performed according to the method described in [57]. Crude mitochondria preparations, obtained from 3-week-old Arabidopsis seedlings, were solubilized with DDM [1.5% (w/v)] and the organellar complexes were resolved by BN-PAGE. For immunodetection, proteins were transferred from the native gels onto a PVDF membrane and were probed with specific antibodies (S2 Table), as indicated below each blot. Arrows indicate to the native complexes I (~1,000 kDa), III (dimer, ~500 kDa), IV (~220 kDa) and V (~600 kDa). The asterisk in the CA2 panel indicates to the presence of a 700 ~ 800 kDa band, which may corresponds to a complex I assembly intermediate. Hybridization signals were analyzed by chemiluminescence assays after incubation with HRP-conjugated secondary antibody. The intensities of protein signals in panels ‘A’ and ‘B’ using ImageJ software [90].

The mtRNA profiles associated with *mterf22* mutants are expected to affect the biogenesis of the respiratory machinery (see e.g., [59, 60, 63, 92]). Accordingly, the translation rates of various plastidial proteins in land-plants were recently shown to be associated with the abundances of their corresponding mRNAs [93]. The accumulation of various mitochondrial proteins in wild-type plants and *mterf22* mutants was studied by immunoblot assays with antibodies raised against different organellar proteins (see S2 Table). These including the complex I (C-I) subunits CA2 (γ-type carbonic anhydrase-like subunit 2) [94] and Nad9 [95], the Rieske iron-sulfur protein (RISP) of complex III (C-III) [96], the cytochrome oxidase subunit 2 (COX2) of complex IV (C-IV), the AtpA subunit of the ATP-synthase enzyme (C-V), the SHMT (serine-hydroxymethyltransferase) protein and the voltage-dependent anion channel (VDAC). These analyses indicated mild reductions (i.e., about 30% to 40%) in the steady-state levels of different mitochondrial proteins, including AtpA, Cox2, Nad9 and RISP (Fig 7A), while the accumulation of CA2, SHMT and VDAC was affected to a lesser degree (2%, 12% and 23% reduced protein signals, respectively) in *mterf22-1* mutants.

We further investigated the biogenesis of the respiratory complexes I, III, IV and V, using BN-PAGEs followed by immunoblot analyses (Fig 7B). The BN-PAGE/immunoblot analyses indicated small reductions (about 25% to 30%) in the levels of native holo-complexes corresponding to C-I, C-III, C-IV and C-V in *mterf22-1* mutant (Fig 7B). We also noticed the presence of a lower molecular weight particle (calculated mass of 700 ~ 800 kDa) corresponding to CA2 protein in *mterf22-1* mitochondria (Fig 7B, and [94]). This protein band (Fig 7B, marked with an asterisk) likely corresponds to a complex I assembly intermediate, as indicated in various Arabidopsis mutants affected in mitochondria gene expression (see e.g., [60, 97–99]). No significant changes in the levels of the chloroplast Rubisco enzyme were seen between the wild-type and mterf22-1 plants (Fig 7B).

### Arabidopsis *mterf22* plants display altered respiratory functions

**Fig 8:**
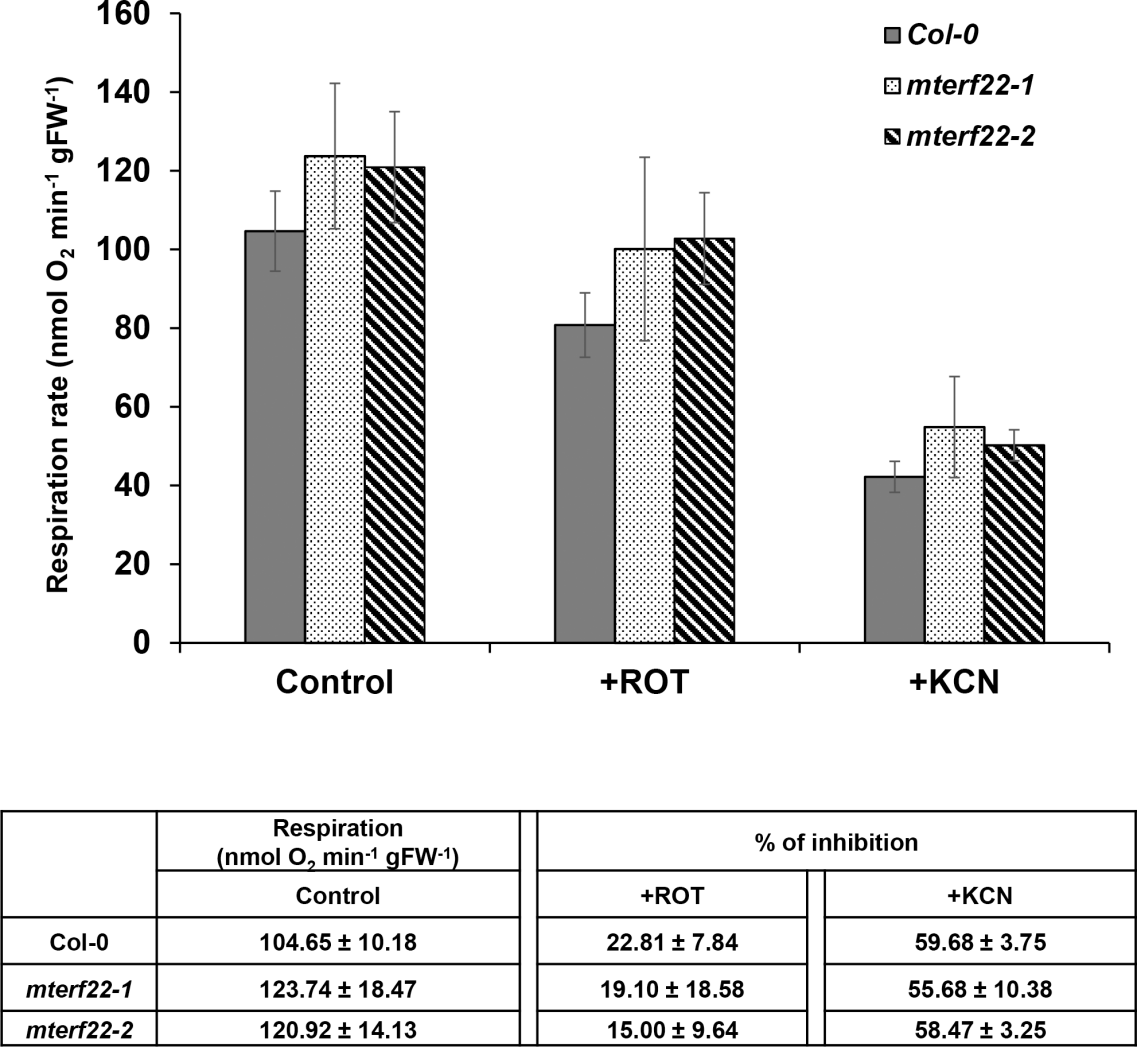
Respiration activities in wild-type and *mterf22* mutants. O_2_-uptake rates of wild-type plants and *mterf22* mutants (i.e., *mterf22-1* and *mterf22-2*) were analyzed with a Clark-type electrode as described previously [60]. For each assay, equal weights (*i*.*e*. 100 mg) of 3-week-old MS-grown Arabidopsis seedlings were submerged in ~2.5 mL sterilized water and applied to the electrode in a sealed glass chamber in the dark. O_2_-uptake rates were measured in the absence (Control) or in presence of rotenone (+ROT, 50 μM) and KCN (1 mM) which inhibit complexes I and IV activities (respectively). The values are means of four biological replicates with about 25 seedlings (i.e., 3-week-old) from each line in each batch. Error bars indicate one standard deviation.

Altered mitochondria morphology and reduced organellar RNA and protein levels in *mterf22* mutants indicate that the functions of mTERF22 are required during mitochondria biogenesis. To determine whether the respiratory activity was also affected in *mterf22* mutants, we monitored the O_2_-uptake rates of 3-week-old wild-type and *mterf22* plants in the dark, using a Clark-type electrode (Fig 8). The average O_2_-uptake rates of wild-type plants was 104.65 ± 10.18 nmol O_2_ min^-1^ gFW^-1^. The O_2_-uptake rates of *mterf22-1* and *mterf22-2* (i.e., 123.74 ± 18.47 and 120.92 ± 14.13 nmol O_2_ min^-1^ gFW^-1^, respectively) were found to be somewhat higher than that of the wild-type plants. Inhibition of complex I activity by 50 μM rotenone (+ROT) had a stronger effect on the average O_2_-upatke rates of wild-type plants (i.e. ~23% inhibition), than on the respiratory activity of *mterf22* plants (i.e., 15% to 19% inhibition) (Fig 8). Similarly, potassium cyanide (+KCN), which specifically inhibits electrons transport through complex IV, may have a stronger effect on the respiration of wild-type plants (~60% reduction in O_2_-uptake rates) than on *mterf22* mutant-lines (55% and 59% inhibition in *mterf22-1* and *mterf22-2*, respectively). However, these assumptions should be considered with care as these mild differences in the respiratory activities between the wild-type and *mterf22* seedlings are not statistically significant.

The increases in the O_2_-uptake rates of *mterf22* mutants (i.e., about 20% higher than the wild-type plants, Fig 8) may correspond to induction of various alternative electron transport pathways in *mterf22-1* (i.e., type II NAD(P)H dehydrogenases, NDs, and alternative oxidases, AOXs), which can bypass the respiration through C-I and C-IV [100–102]. To test this, the expression of various mitochondrial NDs (i.e., NDA1, NDA2, NDB1, NDB2, NDB3, NDB4 and NDC1) and AOXs (i.e., AOX1A, AOX1B, AOX1C, AOX1D and AOX2) was estimated by quantitative RT-PCR. The data revealed to increases in the levels of mRNAs corresponding to both AOX1D and NDA2 in *mterf22* mutants (about 4.5x and 6.0x, respectively; S9 Fig). Accumulation of mRNA was also apparent in the cases of AOX1A, AOX1B, NDA1 and NDB1 (about 2x ~ 3x higher), while the levels of mRNA transcripts corresponding to AOX1C and AOX2 were found similar in the wild-type and mutant plants (S9 Fig). Under the experimental conditions, the expression of NDB2, NDB3, NDB4 and NDC1 was below detectable levels in wild-type and *mterf22-1* plants.

## Discussion

### The *mTERF22* (At5g64950) locus encodes a mitochondrial transcription related factor

The regulation of mtDNA expression is key for controlling the OXPHOS capacity during plant development and in response to various physiological demands. The biogenesis and the regulation of mitochondria gene expression depend upon the activities of numerous nuclear-encoded protein cofactors, which also have the ability to link mitochondrial functions with developmental or environmental signals. However, the identity and functions of mitochondrial transcription factors in plants remain largely unknown. Proteins containing the mTERF domain [27] were found to play important roles in organelle biogenesis in both animals and plants [28, 29, 31, 32]. Interestingly, the mTERF family has been largely extended in angiosperms, however, specific functions have been assigned to only a small number of these proteins. Here, we analyzed the roles of mTERF22 (At5g64950), considering the importance of mTERF proteins in organellar gene expression and RNA metabolism in eukaryotes. GFP localization analyses indicated that mTERF22 resides within the mitochondria in Arabidopsis (Figs 2 and S3, and [28]). Based on microarray datasets, *mTERF22* gene is expressed at low levels in different tissues throughout the plant development (S2 Fig). Analysis of At5g64950 topology indicates that mTERF22 harbors seven repeats of the MTERF domain (Figs 1, S2 and [28]), and may adopts an architecture (i.e., solenoid fold) (S4 Fig) that is structurally related to DNA binding proteins [103].

### mTERF22 is involved in the regulation of mitochondrial gene-transcription in Arabidopsis plants

To analyze the putative roles of mTERF22 in mitochondria gene expression, we examined the growth phenotypes and organellar activities associated with several mutant (T-DNA) lines affected in the At5g64950 gene-locus (i.e., *mterf22-1*, *mterf22-2* and *mterf22-3*; Figs 1 and S2). Under optimal growth conditions (i.e., 22°C) the phenotypes of *mterf22* mutants are generally comparable with those of wild-type plants, but some mild changes in germination rates and seedling development (e.g., shorter roots) are notable when the plants are grown under higher temperatures (i.e., at 28°C; see Fig 3, S4 Table and S5 Fig). Transmission electron microscopy (TEM) further indicated altered mitochondria morphology with less cristae organization in *mterf22* plants (Fig 4). The abnormal morphologies of *mterf22* mutants are correlated with altered transcription of many of the organellar genes in *mterf22* mutants (Fig 5). Genes which their expression is affected (i.e., reduced mRNA steady-state levels) in *mterf22* include *atp1*, *atp4*, *atp6*, *atp8*, *atp9*, *ccmB*, *ccmFc* exons a-b, *ccmFn2*, *cob*, *cox1*, *cox2* exons a-b, *cox3*, *mttB*, *nad1* exons a-b, b-c, c-d, d-e, *nad2* exons a-b, b-c, c-d, d-e, *nad3*, *nad4* exons a-b, b-c, c-d, *nad4L*, *nad5* exons a-b, b-c, c-d, *nad6*, *nad7* exons a-b, b-c, c-d, d-e, *rpl2* exons a-b, *rps4*, *rps7*, *rps12* and *rrn26*. The reduced mRNA levels suggest to altered transcription in *mterf22*, as also indicated by the run-on assays (Fig 6). However, while de-novo organellar transcription was reduced by about 30~50% in *mterf22* mitochondria, the steady-state levels of many mtRNAs were about 4 to 8 times lower in the mutants, *in vivo*. These differences may indicate that altered organellar transcription can also affect the stability or processing of various mtRNAs in *mterf22* mutants.

While the levels of many organellar transcripts is reduced in *mterf22* mutants, the expression of several other genes, including *ccmC*, *ccmFn1*, *rpl16*, *rps3* exons a-b, *nad5* exons d-e and *nad9* transcripts, was not significantly affected in the mutants. RNA-seq data suggest that *nad9*-*nad5de*-*rps3-rpl16* and *ccmC*-*ccmFn1* may be polycistronically expressed (S10 Fig). If true, the transcription of these gene clusters may rely upon transcription factors other than mTERF22. In support of this view, the transcription of *rps3* gene was not significantly affected in *mterf22* mutants (Fig 6). Genetic and biochemical data led to the identification and characterization of many promoter regions in plant mitochondria [17, 24, 104, 105]. Some of the promoter regions contain short sequence motifs, as the YRTA sequence, which may control transcription initiation in plant mitochondria [17, 24, 104, 106]. However, a comparative sequence analyses of the upstream regions of predicted polycistronic units in Arabidopsis mitochondria (S10 Fig) failed to indicate common sequence motifs that could explain the differences in gene expression seen in *mterf22*. It was previously noted that RpoTm could accurately initiate organellar transcription of many promoter regions in the absence of auxiliary factors, in vitro. However, it remains possible that for efficient transcription in vivo, RpoTm relies on the activities of various transcription factors, which may stabilize open promoter complexes or assist with the release of the polymerase following RNA synthesis [24]. Biochemical analysis of mTERF22 binding to different promoter regions and its possible association to RpoTm would be required to test this intriguing hypothesis.

### Mutations in mTERF22 lead to mitochondrial oxidative phosphorylation defects

The respiratory machinery is composed of four major electron transport complexes (C-I to C-IV) and the ATP synthase (also denoted as C-V), which are required for cell respiration and aerobic energy production [102, 107]. The biogenesis of the respiratory machinery depends upon a concerted expression of both nuclear- and organellar-encoded subunits [10, 13, 102]. Altered transcription in *mterf22* (Figs 4 and 5) may affect the organellar translation and thereby to alter respiratory-mediated functions. While many organellar transcripts were reduced by about 4 to 8 folds in *mterf22*, proteomic studies indicated to only mild reductions in the accumulation of various organellar proteins (Fig 7A) in *mterf22*. Regulation of gene transcription is key to gene expression response. While the translation of some proteins fine‐tune the transcription levels, the steady-state levels of many other proteins poorly correlate with the levels of their corresponding mRNAs [108–110]. Accordingly, the biogenesis of the respiratory machinery was only mildly affected (Figs 7 and 8) in the mutants. Small increases in the O_2_-uptake rates of the mutants (Fig 8) may correspond to the induction of the alternative respiratory pathway in *mterf22* (i.e., higher AOX1D and NDA2 transcript levels, S10 Fig).

### The phenotypes of *mterf22* mutants

Our data indicate that the *mterf22* mutants are able to grow, flower and set viable seeds. Under the optimum growth temperature range for Arabidopsis (i.e., 22~23°C; ABRC, https://abrc.osu.edu/), the homozygous *mterf22* lines exhibit normal phenotypes, although some growth and developmental defects (i.e., reduced germination and short root phenotypes) can be observed when the plants are grown at 28°C (Figs 3 and S5). We assume that these phenotypes correspond to reduced plant fitness under the stress conditions, as the global mtRNA profiles of *mterf22* mutants grown at 22°C (see S11 Fig) are similar to those of the mutant plants grown at 28°C (Fig 5). In light of the expected significance of mitochondria function to plant physiology, why does the loss of mTERF22 cause such little phenotypic effects? Currently, we cannot provide a definitive explanation, but we speculate that the lack of strong phenotypes, associated with *mterf22* mutants, are the consequences of redundant functions between different transcription factors that may exist in plant mitochondria. These may involve other mTERFs (e.g., mTERF7 and mTERF27), which are phylogenetically related and share significant sequence similarity with mTERF22 (S8 Fig), or any other transcription factors still awaiting their functional characterization. This assumption is strongly supported by the fact that the mutations in *mTERF22* gene have not abolish the transcription in *mterf22* mutants (Figs 5 and 6). RT-qPCR analyses indicate that the mRNA levels of mTERF7 or mTERF27 was not significantly changed in *mterf22* mutants (S12 Fig), however, these proteins may be upregulated in their expression or activities in the *mterf22* mutants.

### Conclusions

Mitochondria play pivotal roles in cellular energy metabolism. Specific changes in the rates of respiration are required to meet alterations in energy demands during particular stages in plant growth and development, or under different environmental conditions. The regulation of organellar biogenesis and activity is mediated by numerous nuclear-encoded cofactors that await to be deciphered. The roles of mTERF proteins in the regulation of mitochondria gene expression in plants is particularly relevant because they have been implicated in organellar transcription, translation and DNA replication in different eukaryotes. Our work indicates that mTERF22 is involved in mtDNA transcription in Arabidopsis. Mutants in *mTERF22* gene-locus are only partially affected in the biogenesis of the respiratory machinery and exhibit normal phenotypes under optimal growth conditions (i.e., at 22°C), but seem to show some mild defects in growth and developmental under mild stress conditions (i.e., at 28°C). These data coincide with lower steady-state levels of many mtRNAs in *mterf22* mutants. We speculate that these phenotypes may be due to functional redundancies between mTERF22 and other transcription factors in Arabidopsis mitochondria. The examination of this hypothesis would require the establishment of mutants affected in different mTERFs. Studies are under way in our laboratory to investigate the roles of mitochondrial mTERFs in Arabidopsis.

## Supporting information

S1 Table List of primers used in gene expression studies, GFP localizations analyses and mutant plants screening. (PDF)

S2 Table List of antibodies used for the analysis of *mterf22* mutants. (PDF)

S3 Table List of primers used in mitochondria transcriptome and splicing analyses by RT-qPCR. (PDF)

S4 Table Germination rates in wild-type (Col-0) and mutant plants. (PDF)

S1 Fig Differential expression of mTERF7, mTERF22 and mTERF27 in Arabidopsis thaliana (col-0) plants. **(**A) Analysis of the expression profiles of mTERF22 (encoded by the At5g64950 gene-locus), available in the Genevestigator [65] and AtGeExpress [66] microarray databases, show that mTERF22 is expressed at low levels in different tissues throughout the plant’s development. **(**B) Differential expression of mTERF7 (At5g07900), mTERF22 and mTERF27 (At1g21150) in *Arabidopsis thaliana* (Col-0) plants. The relative steady-state levels of mRNAs corresponding to mTERF7, mTERF22 and mTERF27 was determined by RT-qPCR in 3 week-old wild-type, *mterf22-1* and *mterf22-2* plants after normalization to the *actin2* (At3g1878) and 18S rRNA (At3g41768) genes (see S1 Table) [59–61, 63, 81]. The values are mean of three independent biological replicates, using 35~50 seedlings from each line in each assay. Error bars indicate one standard deviation).
(TIF)

S2 Fig Arabidopsis *mTERF22* gene structure. The nucleotides (A) and amino acids (B) sequences of mTERF22. Underlined letters indicate to the 5’ and 3’ untranslated regions (UTRs), as indicated by the RACE analysis and TAIR database, while uppercased letters represent the open reading frame of mTERF22. The position of T-DNA insertions in *mterf22* mutants i.e., *mterf22-1* (SALK- 032680), *mterf22-2* (SAIL-1228) and *mterf22-3* (SALK-133048) are indicated by red triangles. Panel B represents the amino acid sequence of Arabidopsis mTERF22 protein. The postulated regions corresponding to the mitochondrial targeting sequence (21 amino acid long, underlined and highlighted in blue) and the seven MTERF motifs (highlighted in magenta) of mTERF22 were predicted by the TargetP and SMART servers.
(TIF)

S3 Fig Analysis of the intracellular locations of GFP fusion proteins in tobacco cells. Tobacco plants were transformed with GFP alone (panels A to D) or GFP fused to the N-termini region (about 150 amino acids) of ATP synthase b-subunit (panels E to H). GFP signals (green, upper left, panels A and E), MitoTracker marker (red, upper right, panels B and F), chlorophyll autofluorescence (blue, lower left, panels C and G) and merged images (lower right, panels D and H), are outlined in each panel. The position of the nucleus (N) is indicated in panels A and E.
(TIF)

S4 Fig Structural model of *Arabidopsis thaliana* mTERF22 protein. Schematic representation of the putative 3D structure of Arabidopsis mTERF22 protein. To get more of an insight on mTERF22's mode of action, in particular of DNA recognition, we performed an atomic model of the protein using the Phyre server (Kelley and Sternberg 2009). The model structure of mTERF22 (*i*.*e*. ribbon and surface views) were generated by the PyMol software suite [77]. A, B and C represent the same structure from different angles. Similarly to the mammalian mTERF1 and mTERF3 proteins [74–76], the predicted 3D structure of mTERF22 suggested a solenoid-like fold [103]. The color code is red for negative values, white for near zero values, and blue for positive values. Positively charged surfaces are expected to be critical for nucleotide recognition and binding, while uncharged or positively charged regions may function in protein-protein interactions.
(TIF)

S5 Fig Plant phenotypes associated with mTERF22 mutations grown at 28°C. The effects of *mTERF22*-suppression on the growth phenotypes and development of Arabidopsis wild-type (Col-0) and *mterf22* knockout lines. (A) Growth phenotypes associated with 3-week-old wild-type and homozygous *mterf22* seedlings grown on MS-agar plates at 28°C. (B) 2-week-old wild-type and *mterf22* seedlings grown vertically on MS-agar plates at 28°C. (C) The average root lengths of wild-type and *mterf22* mutants grown at 28°C. The values are means of three biological replicates with ~30 seedlings from each line. Error bars indicate one standard deviation. Statistical significance was set at P < 0.05.
(TIF)

S6 Fig The suppression of mTERF22 has only a minor effect on the splicing efficiencies of various mitochondrial group II introns. Quantitative RT-PCR of unspliced (pre-mRNA) and spliced (mRNA) mitochondrial transcripts in wild-type and *mterf22-1* plants, was preformed as described in Zmudjak et al. (2017), after normalization to the *actin2* (At3g1878), and 18S rRNA (At3g41768) genes. The histogram shows the ratios of pre-RNAs to mRNA between *mterf22* and wild-type plants. The values are means of four biological replicates using 35~50 seedlings from each line in each assay. Error bars indicate one standard deviation.
(TIF)

S7 Fig DNA copy numbers in wild-type (Col-0) and *mterf22* plants. Relative mtDNA copy numbers in *mterf22-1* mutants versus wild-type plants were analyzed by qPCR with oligonucleotides designed to different mitochondrial genes. Primers used in the qPCR analyses are listed in Supplemental S4 Table. The values are means of five biological replicates, using 35~50 seedlings from each line in each assay. Error bars indicate one standard deviation.
(TIF)

S8 Fig Phylogenetic analysis of Arabidopsis mTERF family. (A) Phylogeny tree was constructed using the T-Coffee multiple sequence alignment server [71], with the 35 known Arabidopsis mTERF protein sequences (bootstrap values for 1,000 bootstrap replicates). The scale bar represents the number of amino acid substitutions per site. (B) Alignment of mTERF7, mTERF22 and mTERF27 was conducted with T-Coffee multiple sequence alignment server, and displayed using GeneDoc [72] with the conserved residue shading mode.
(TIF)

S9 Fig Relative accumulation of alternative oxidases (AOXs) and type II NAD(P)H dehydrogenases (NDs) in wild-type (WT) and *mterf22* mutant plants. Relative quantification of nuclear-encoded genes related to the alternative respiration pathway in the wild type and *mterf22* mutants. The relative steady-state levels of mRNAs corresponding to various AOXs and alternative NAD(P)H isoforms was determined by RT-qPCR in 3 week-old wild-type, *mterf22-1* and *mterf22-2* plants after normalization to the *actin2* (At3g1878) and 18S rRNA (At3g41768) genes [59–61, 63, 81]. The values are mean of three independent biological replicates, using 35~50 seedlings from each line in each assay. Error bars indicate one standard deviation.
(TIF)

S10 Fig Transcriptome mapping of Arabidopsis mitochondria by RNA-seq analysis. Total mtRNA was extracted from Arabidopsis mitochondria. RNA-sequencing (RNA-seq) was carried out on Illumina Genome Analyzer (The Genome Laboratory, The Hebrew University, Jerusalem, Israel), essentially as described previously [7, 61]. Data are shown for the regions encompassing the supposed polycistronic units of *nad5-nad9-rps3* (A) and *ccmC-ccmFn1* (B) gene clusters. Green, blue and red lines point to sequence variations (typically C-to-U RNA editing) between the mtRNA-seq data and the mtDNA of Arabidopsis (NC_001284; [84]). Black arrows indicate to the direction of transcription.
(TIF)

S11 Fig Transcript abundance of mitochondrial mRNAs in Arabidopsis wild-type and *mterf22-1* plants grown at 22°C. The accumulation of various mtRNAs in wild-type and *mterf22* plants was analyzed by quantitative reverse transcription PCR (RT-qPCR). RNA extracted from 3-week-old wild-type (Col-0) and *mterf22-1* mutant plants grown at 22°C was reverse-transcribed, and the relative steady-state levels of different organellar transcripts were evaluated by qPCR with specific oligonucleotides, after normalization to the *actin2* (At3g1878), 18S rRNA (At3g41768) and 26S rRNA (Atmg00020) genes (see S1 and S3 Tables). The histogram shows the relative mRNAs levels (*i*.*e*. log2 ratios) in mutant lines versus those of wild-type plants. The values are means of three biological replicates, using about 35~50 seedlings from each line in each assay. Error bars indicate one standard deviation.
(TIF)

S12 Fig Transcript abundance of mitochondrial mTERF7 and mTERF27 in Arabidopsis wild-type (Col-0) versus *mterf22* mutants plants. The accumulation of transcripts corresponding to mTERF7 and mTERF27 in wild-type (Col-0) and *mterf22* plants was analyzed by quantitative reverse transcription PCR (RT-qPCR). RNA extracted from 3-week-old wild-type (Col-0) and *mterf22* mutants plants grown at 22°C was reverse-transcribed, and the relative abundances (i.e., steady-state levels) of *mTERF7* (AT5G07900) and *mTERF27* (AT1G21150) transcripts were evaluated by qPCR with specific oligonucleotides, after normalization to the *actin2* (At3g1878), 18S rRNA (At3g41768) and 26S rRNA (Atmg00020) genes (see S1 Table). The histogram shows the relative mRNAs levels in mutant lines versus those of the wild-type plants. The values are means of three biological replicates, using about 30 Arabidopsis seedlings. Error bars indicate one standard deviation.
(TIF)
