# Correction: Control of organelle gene expression by the mitochondrial transcription termination factor mTERF22 in *Arabidopsis thaliana* plants

**DOI:** 10.1371/journal.pone.0214558

**Published:** 2019-03-25

**Authors:** Sofia Shevtsov, Keren Nevo-Dinur, Lior Faigon, Laure D. Sultan, Michal Zmudjak, Mark Markovits, Oren Ostersetzer-Biran

After publication of this article [1], concerns were raised about the following:

- There are two horizontal discontinuities in the CA2 (C-I) blot in Fig 7B, one identified by a difference in background and the other by a thin line.
- The GFP marks in Fig 1A do not correspond to the mitotracker marks in Fig 2B, which undermines the conclusions about the localization of mTERF22 protein (encoded by the At5g64950 gene-locus).

The authors acknowledge that horizontal irregularities are visible in the image at a high exposure. The first irregularity noted is the lighter background seen in the lower part of the gel (below arrow 1 in panel B and below arrow 3 in panel D). This corresponds to the background that the PVDF membrane was placed on for the imaging and does not affect the conclusions in any way.

The second irregularity noted is a horizontal white line in the CA2 blot in Fig 7B. This irregularity is not observed in the original blots or the original version of the figure. In a discussion with the *PLOS ONE* staff and a member of the *PLOS ONE* Editorial Board, the authors concluded that this line does not affect the conclusions and was likely an artefact during conversion between file types.

The original blot can be viewed via Figshare at https://figshare.com/articles/PLOS-One_Original_files/7207547. The revised Fig 7 is included here.

**Fig 7:**
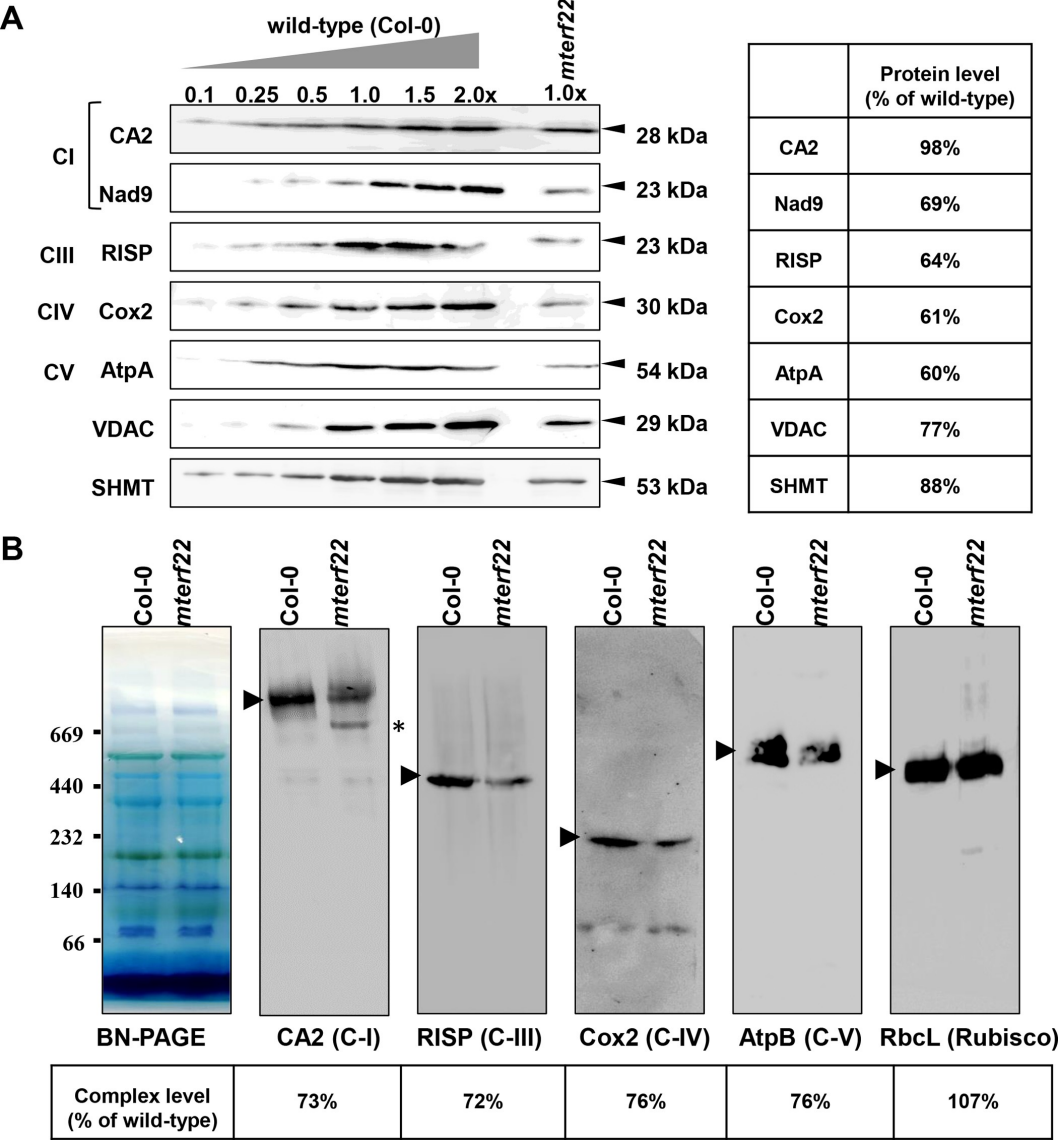
Relative accumulation of organellar proteins in wild-type and mterf22 plants. (A) Immunoblot analyses of wild-type plants and *mterf22-1* mutant line. For the quantification of the relative abundances of organellar proteins in *mterf22* plants, different amounts of total mitochondrial proteins extracted from wild-type plants were loaded and separated by SDS-PAGE. The blots were probed with polyclonal antibodies raised to different organellar proteins, as indicated in each panel. Detection was carried out by chemiluminescence assays after incubation with HRP-conjugated secondary antibody. (B) BN-PAGE of crude mitochondria preparations was performed according to the method described in [57]. Crude mitochondria preparations, obtained from 3-week-old Arabidopsis seedlings, were solubilized with DDM [1.5% (w/v)] and the organellar complexes were resolved by BN-PAGE. For immunodetection, proteins were transferred from the native gels onto a PVDF membrane and were probed with specific antibodies (S2 Table), as indicated below each blot. Arrows indicate to the native complexes I (~1,000 kDa), III (dimer, ~500 kDa), IV (~220 kDa) and V (~600 kDa). The asterisk in the CA2 panel indicates to the presence of a 700 ~ 800 kDa band, which may correspond to a complex I assembly intermediate. Hybridization signals were analyzed by chemiluminescence assays after incubation with HRP-conjugated secondary antibody. The intensities of protein signals in panels ‘A’ and ‘B’ using ImageJ software [90].

In regard to the conclusions about the localization of mTERF22 protein (encoded by the At5g64950 gene-locus), the authors agree that, based on the GFP-data, the authors cannot confirm that mTERF22 is exclusively located within the mitochondria, particularly, as the GFP fusion construct contains only a portion (100 amino acids of the N-termini part) of mTERF22 protein. However, the authors would also like to note that in the original images (available now in Figshare), the GFP signals correspond to most of the mitotracker marks in Fig 2B, although the mito-marker produced weak intensities in this region (i.e., right side of the image). Taken together with the GFP-localization data in Fig 2, the in silico localization predictions (SUBA database), and the localization data shown in Babiychuk et al, 2011 (Supplementary Figures S1 and S2, DOI: 10.1073/pnas.1103442108), these results clearly support that mTERF22 resides within the mitochondria in Arabidopsis and tobacco plants.

In addition, the primary data underlying results in this article were not included with the published article although the Data Availability Statement for this article stated, “All relevant data are within the paper and its Supporting Information files.” With this Correction, the authors provide the original raw data via Figshare at https://figshare.com/articles/PLOS-One_Original_files/7207547.

The authors have provided the original raw data for this experiment which support the results and conclusions as reported in the published article.
