# Supplementary material for: Control of organelle gene expression by the mitochondrial transcription termination factor mTERF22 in Arabidopsis thaliana plants
Source: PLoS One. 2018 Jul 30;13(7):e0201631. doi: 10.1371/journal.pone.0201631 (PMC6066234; doi:10.1371/journal.pone.0201631)
Supplement: S1 Table — (PDF) [file pone.0201631.s001.pdf]

S1 Table. List of primers used in gene expression studies, GFP localizations analyses and mutant plants screening.

Target gene	oligo name	oligonucleotide sequence (5'-to-3')
mterf22 (At5g64950)	*mterf22F*	ATCCAGCGTTCGTCGAGTTT
	mterf22R	AGCAAGCTGAGACCCAACAA
mterf22 GFP-localization assay	*NcoInewF*	CAAGATCCATGGTACAATTTCTAGCTTCTCTCGTTT
	BamHInewR	GATATAGGATCCCGAACATCATTCGAGGATGGA
	Sat6-EcorIF	ACATAGGAATTCTTCCCAGTCACGACGTTGTA
	Sat6-SmaIR	ACATAGCCCGGGCAGCTATGACCATGATTACTG
T-DNA screening	*mterf22.1R*	AGCAAGCTGAGACCCAACAA
	mterf22.2F	GTTTGGTCGGAGATGGGAT
	mterf22.3R	GAGAGACGAACTTACCAAGA
	LBb1.3 (SALK)	ATTTTGCCGATTTCGGAAC
	LB3 (SAIL)	GCCTTTTCAGAAATGGATAAATAGCCTTGCTTCC
actin2 (AT3G18780)	*actin2-F*	GGTAACATTGTGCTCAGTGGTGG
	actin2-R	AACGACCTTAATCTTCATGCTGC
mTERF7 (At5g07900)	*mterf7-F*	AGGATTATCCCGAGGTGTTCGG
	mterf7-R	ATCCTCTTTCACAAGCCCGTTCG
mTERF22 (At5g07900)	*mterf22-F*	TGTCTCTCGTGCTTTGGATCTTGG
	mterf22-R	AACAGCGTGCACAAGCATCCTC
mTERF27 (At5g07900)	*mterf27-F*	ACCATGGCTTCACCAATGACCAG
	mterf27-R	TACATCTTCCGGGCTCAGTGAC
AOX2 (At5g64210)	*AOX2-F*	ACGGTGATTCGTGCTGATGAAGC
	AOX2-R	TCCTTGATTGCGAATGTCAGAAGC
AOX1A (At3g22370)	*AOX1A -F*	AGCATCATGTTCCAACGACGTTTC
	AOX1A -R	GCTCGACATCCATATCTCCTCTGG
AOX1B (At3g22360)	*AOX1B -F*	GGACCGTGAAATCTCTTCGATGGC
	AOX1B -R	TCTAGCATCATTGCTCTGCATCCG
AOX1C (At3g27620)	*AOX1C -F*	TCTTCCAGAGGAGGTATGGTTGCC
	AOX1C -R	AGTGCATAAGCATCCCTCCAACC
AOX1D (At1g32350)	*AOX1D -F*	TTTGCTCGAAGAGGCTGAGAACG
	AOX1D -R	CTCGTTCGTACCATTTGGGTTGTG
NDA1 (At1g07180)	*NDA1 -F*	GTATCCAACCGGCGATTTCACG
	NDA1 -R	AGTTACAGTCTCACAATGCACCTC
NDA2 (At2g29990)	*NDA2 -F*	TGGTGTTGGTCCTTCTCCTTTCG
	NDA2 -R	TCCATTCGTCAATGCCAATCCTTC
NDB1 (At4g28220)	*NDB1 -F*	TAACACATTTGGCACTCCTGGTG
	NDB1 -R	CTCTGTGCATCCTCTACTTCCTTG
NDB2 (At4g05020)	*NDB2 -F*	AGGAGGAGAGGTTTCCTCGATTC
	NDB2 -R	CAGAGCTCGTCTATTACCCTGACC
NDB3 (At4g21490)	*NDB3 -F*	TCCAGGAGACTGGGTTTCGATAGG
	NDB3 -R	GCCAGCTCACTTGCTTACTAGC
NDB4 (At2g20800)	*NDB4 -F*	GTGGTCAACAGGAATTGGTTCACG
	NDB4 -R	AAGACACGTCTCTGTCCCTGAC
NDC1 (At5g08740)	*NDC1 -F*	CGAGAGCAGTGACAAACAACAGTG
	NDC1 -R	AATGCACACCCTTGGCCTCTTG
