# Supplementary material for: Control of organelle gene expression by the mitochondrial transcription termination factor mTERF22 in Arabidopsis thaliana plants
Source: PLoS One. 2018 Jul 30;13(7):e0201631. doi: 10.1371/journal.pone.0201631 (PMC6066234; doi:10.1371/journal.pone.0201631)
Supplement: S2 Table — (PDF) [file pone.0201631.s002.pdf]

S2 Table. List of antibodies used for the analysis of *mterf22* mutants.

Antibody	Protein I.D.	origin	serum	dilution	Reference / source
AtpA	Mitochondrial ATP-synthase subunit α	*Zea mays*	Mouse (monoclonal)	1/5,000	(Michael *et al.* 1993)
AtpB	Mitochondrial ATP-synthase subunit β	*Zea mays*	Mouse (monoclonal)	1/5,000	(Michael *et al.* 1993)
CA2	γ-carbonic anhydrase-like subunit 2	*Arabidopsis thaliana*	Rabbit (polyclonal)	1/1,000	(Perales *et al.* 2005, Sunderhaus *et al.* 2006)
Cox2	Cytochrome oxidase subunit-2	*Arabidopsis thaliana*	Rabbit (polyclonal)	1/5,000	Agrisera antibodies, AS04 053A
Nad9	NADH-dehydrogenase complex subunit-9	*Triticum spp.*	Rabbit (polyclonal)	1/50,000	(Lamattina *et al.* 1993)
RISP	Rieske iron-sulfur protein	*Arabidopsis thaliana*	Rabbit (polyclonal)	1/5,000	Gift of Prof. Ian Small, UWA
RbcL	Large subunit of chloroplast Rubisco enzyme	*Pisum sativum*	Rabbit (polyclonal)	1/1,000	Gift of Prof. Michal Shapira, BGU
SHMT1	Serine hydroxymethyltransferase 1	*Spinacia oleracea*	Rabbit (polyclonal)	1/1,000	Agrisera antibodies, AS05 075
Porin (VDAC)	Voltage-dependent anion channel	*Zea mays*	Mouse (monoclonal)	1/5,000	Thomas Elthon collection, PM035

References

Keren, I., Bezawork-Geleta, A., Kolton, M., Maayan, I., Belausov, E., Levy, M., Mett, A., Gidoni, D., Shaya, F. and Ostersetzer-Biran, O. (2009) AtnMat2, a nuclear-encoded maturase required for splicing of group-II introns in Arabidopsis mitochondria. *RNA*, **15**, 2299-2311.

Lamattina, L., Gonzalez, D., Gualberto, J. and Grienenberger, J.-M. (1993) Higher plant mitochondria encode an homologue of the nuclear-encoded 30-kDa subunit of bovine mitochondrial complex I. *Eur J Biochem*, **217**, 831-838.

Matthes, A., Schmidt-Gattung, S., Köhler, D., Forner, J., Wildum, S., Raabe, M., Urlaub, H. and Binder, S. (2007) Two DEAD-Box Proteins May Be Part of RNA-Dependent High-Molecular-Mass Protein Complexes in Arabidopsis Mitochondria. *Plant Physiol*, **145**, 1637-1646.

Michael, H.L., Arnost, H. and Elthon, T.E. (1993) Monoclonal Antibodies to the a- and b- Subunits of the Plant Mitochondrial F_1-ATPase. *Plant Physiology*, **101**, 931-937.

Perales, M., Eubel, H., Heinemeyer, J., Colaneri, A., Zabaleta, E. and Braun, H.-P. (2005) Disruption of a nuclear gene encoding a mitochondrial gamma carbonic anhydrase reduces complex I and supercomplex I+III2 levels and alters mitochondrial physiology in Arabidopsis. *J Mol Biol*, **350**, 263-277.

Sunderhaus, S., Dudkina, N.V., Jansch, L., Klodmann, J., Heinemeyer, J., Perales, M., Zabaleta, E., Boekema, E.J. and Braun, H.P. (2006) Carbonic anhydrase subunits form a matrix-exposed domain attached to the membrane arm of mitochondrial complex I in plants. *J Biol Chem*, **281**, 6482-6488.
