# Supplementary material for: Control of organelle gene expression by the mitochondrial transcription termination factor mTERF22 in Arabidopsis thaliana plants
Source: PLoS One. 2018 Jul 30;13(7):e0201631. doi: 10.1371/journal.pone.0201631 (PMC6066234; doi:10.1371/journal.pone.0201631)
Supplement: S3 Table — (PDF) [file pone.0201631.s003.pdf]

S3 Table. List of primers used in mitochondria transcriptome and splicing analyses by RT-qPCR.

gene target	oligo name	sequence (5'-to-3')
AOX1A	*aox1aF*	AGCATCATGTTCCAACGACGTTTC
	aox1aR	GCTCGACATCCATATCTCCTCTGG
AOX1B	*aox1bF*	GGACCGTGAAATCTCTTCGATGGC
	aox1bR	TCTAGCATCATTGCTCTGCATCCG
AOX1C	*aox1cF*	TCTTCCAGAGGAGGTATGGTTGCC
	aox1cR	AGTGCATAAGCATCCCTCCAACC
AOX1D	*aox1dF*	TTTGCTCGAAGAGGCTGAGAACG
	aox1dR	CTCGTTCGTACCATTTGGGTTGTG
AOX2	*aox2F*	ACGGTGATTCGTGCTGATGAAGC
	aox2R	TCCTTGATTGCGAATGTCAGAAGC
NDA1	*nda1F*	GTATCCAACCGGCGATTTCACG
	nda1R	AGTTACAGTCTCACAATGCACCTC
NDA2	*nda2F*	TGGTGTTGGTCCTTCTCCTTTCG
	nda2R	TCCATTCGTCAATGCCAATCCTTC
NDB1	*ndb1F*	TAACACATTTGGCACTCCTGGTG
	ndb1R	CTCTGTGCATCCTCTACTTCCTTG
atp1	*atp1F*	TCACTTCGACACGTCTTTGC
	atp1R	GGAATGGCCTTGAATCTTGA
atp6	*atp6-1F*	TCTTTTGCGAGTCAATGCAC
	atp6-1R	TCTCGCGTATCTCACATTGC
atp8	*atp8F*	CCGTCGACTTATTGGGAAAA
	atp8R	TTCCTTGGCCATGTACAACA
atp9	*atp9F*	CATTCCCTCTGACGTCGAAT
	atp9R	TCGTCGATTCTTACCCTCGT
atp4	*atp4F*	GGATCAGCTTGCGAATTTGT
	atp4R	GCAAATTGCTTCCCCACTAA
ccmb	*ccmBF*	TCTTGGAATCACATCCAGCA
	ccmBR	CGAGACCGAAATTGGAAAAA
ccmc	*ccmCF*	AGCTACGCGCAAATTCTCAT
	ccmCR	GCCGTGGCGATATAAACAAT
ccmfc	*ccmFcF*	CACATGGAGGAGTGTGCATC
	ccmFcR	GTGGGTCCATGTAAATGATCG
ccmfn-1	*ccmFN1F*	AGCTCTTGGCATTGCTTTGT
	ccmFN1R	AGTGCCACAATCCCATTCAT
ccmfn-2	*ccmFN2F*	CGTGTCGTTCGTAATGGAAA
	ccmFN2R	TGATAAGCCCACCAACTTCC
cob	*cobF*	TGCCGGAATGGTATTTCCTA
	cobR	GCCAAAAGCAACCAAAACAT
cox1	*cox1F*	GTAGCTGCGGTGAAGTAGGC
	cox1R	CTGCCTGGATTCGGTATCAT

cox2	*cox2F*	TGATGCTGTACCTGGTCGTT
	cox2R	TGGGGGATTAATTGATTGGA
cox3	*cox3F*	CCGTAACTTGGGCTCATCAT
	cox3R	AAACCATGAAAGCCTGTTGC
mttb	*mttBF*	GGGGTCTTTCTTTGGAAACC
	mttBR	TCTCCCTCATTCCACTCGTC
nad1 exons a-b	*nad1 1-2F*	GACCAATAGATACTTCATAAGAGACCA
	nad1 1-2R	TTGCCATATCTTCGCTAGGTG
nad1 exons b-c	*nad1 2-3F*	ATTCAGCTTCCGCTTCTGG
	nad1 2-3R	TCTGCAGCTCAAATGGTCTC
nad1 exons c-d	*nad1 3-4F*	AAAAGAGCAGACCCCATTGA
	nad1 3-4R	TCCGTTTGATCTCCCAGAAG
nad1 exons d-e	*nad1 4-5F*	AGCCCGGGATCTTCTTGA
	nad1 4-5R	TCTTCAATGGGGTCTGCTC
nad2 exons a-b	*nad2 exons a-bF*	GCGAGCAGAAGCAAGGTTAT
	nad2 exons a-bR	GGATCCTCCCACACATGTTC
nad2 exons b-c	*nad2 exons b-cF*	AAAGGAACTGCAGTGATCTTGA
	nad2 exons b-cR	AATATTTGATCTTAGGTGCATTTTC
nad2 exons c-d	*nad2 exons c-dF*	GCGCAATAGAAAGGAATGCT
	nad2 exons c-dR	CTATGGGTCTACTGGAGCTACCC
nad2 exons d-e	*nad2 exons d-eF*	CAAAGGAGAGGGGTATAGCAA
	nad2 exons d-eR	TATTTGTTCTTCGCCGCTTT
nad3	*nad3F*	CGAATGTGGTTTCGATCCTT
	nad3R	GCACCCCTTTTCCATTCATA
nad4 exons a-b	*nad4 exons a-bF*	ATTCTATGTTTTTCCCGAAAGC
	nad4 exons a-bR	GAAAAACTGATATGCTGCCTTG
nad4 exons b-c	*nad4 exons b-cF*	AATACCCATGTTTCCCGAAG
	nad4 exons b-cR	TGCTACCTCCAATTCCCTGT
nad4 exons c-d	*nad4 exons c-dF*	TTCCTCCATAAATTCTCCGATT
	nad4 exons c-dR	TGAAATTTGCCATGTTGCAC
nad4L	*nad4L-F*	GGGGAATCCTCCTTAATAGACG
	nad4L-R	AACGAAAATGGCTAACCCAATA
nad5 exons a-b	*nad5 exons a-bF*	TGGACCAAGCTACTTATGGATG
	nad5 exons a-bR	CCATGGATCTCATCGGAAAT
nad5 exons b-c	*nad5 exons b-cF*	TACCTAAACCAATCATCATATC
	nad5 exons b-cR	CTGGCTCTCGGGAGTCTCTT
nad5 exons c-d	*nad5 exons c-dF*	AACTCGGATTCGGCAAGAA
	nad5 exons c-dR	GATATGATGATTGGTTTAGGTA
nad5 exons d-e	*nad5 exons d-eF*	AACATTGCAAAGGCATAATGA
	nad5 exons d-eR	GTTCCTGCGTTTCGGATATG
nad6	*nad6F*	TATGCCGGAAAGGTACGAAG
	nad6R	GTGAGTGGGTCAGTCGTCCT
nad7 exons a-b	*nad7 exons a-bF*	ACCTCAACATCCTGCTGCTC
	nad7 exons a-bR	AAGGTAAAGCTTGAAGATAAGTTTTGT

nad7 exons b-c	*nad7 exons b-cF*	GAGGGACTGAGAAATTAATAGAGTACA
	nad7 exons b-cR	TGGTACCTCGCAATTCAAAA
nad7 exons c-d	*nad7 exons c-dF*	ACTGTCACTGCACAGCAAGC
	nad7 exons c-dR	CATTGCACAATGATCCGAAG
nad7 exons d-e	*nad7 exons d-eF*	GATCAAAGCCGATGATCGTAA
	nad7 exons d-eR	AGGTGCTTCAACTGCGGTAT
nad9	*nad9F*	GGATGACCCTCGAAACCATA
	nad9R	CACGCATTCGTGTACAAACC
rpl2	*rpl2F*	CCGAAGACGGATCAAGGTAA
	rpl2R	CGCAATTCATCACCATTTTG
rpl5	*rpl5F*	AAGGGGTTCGACAGGAAAGT
	rpl5R	CGTATTTCGACCGGAAAATC
rpl16	*rpl16F*	GAGCATTTGCCAAACTCACA
	rpl16R	CGGACACTTTCATCGTGCTA
rps3	*rps3F*	CCGATTTCGGTAAGACTTGG
	rps3R	AGCCGAAGGTGAGTCTCGTA
rps4	*rps4F*	ACCCATCACAGAGATGCACA
	rps4R	TCACACAAACCCTTCGATGA
rps7	*rps7F*	CTCGAACTGAACGCGATGTA
	rps7R	AAGCTGCTTCAAGGATCCAA
rps12	*rps12F*	AGCCAAAGTACGGTTGAGCA
	rps12R	TTTGGGTTTTTCTGCACCAT
rrn18	*rrn18F*	CGTCACCTGGGTCAAAAACT
	rrn18R	GCTTGAAAACCGAAGTGAGC
rrn26	*rrn26F*	GACGAGACTTTCGCCTTTTG
	rrn26R	CTTGGAGCGAATTGGATGAT
18S rRNA (At3g41768)	*18S nucl-F*	AAACGGCTACCACATCCAAG
	18S nucl-R	ACTCGAAAGAGCCCGGTATT
actin2 (At3g18780)	*actin2-F*	GGTAACATTGTGCTCAGTGGTGG
	actin2-R	AACGACCTTAATCTTCATGCTGC
