# Supplementary material for: Control of organelle gene expression by the mitochondrial transcription termination factor mTERF22 in Arabidopsis thaliana plants
Source: PLoS One. 2018 Jul 30;13(7):e0201631. doi: 10.1371/journal.pone.0201631 (PMC6066234; doi:10.1371/journal.pone.0201631)
Supplement: S4 Table — (PDF) [file pone.0201631.s004.pdf]

S4 Table. Germination rates in wild-type (Col-0) and mutant plants

mutant lines	22ºC		28ºC	
	Number of germinating seeds	Germination rates (%)	Number of germinating seeds	Germination rates (%)
Col-0	242/243	99.6	79/87	90.1
*nmat1**[1]	161/211	76.3	95/124	76.6
mterf22-1 heterozygote line	204/208	98.1	59/95	90.8
mterf22-1 homozygous line	207/208	99.5	83/106	78.3
mterf22-2 homozygous line	124/127	97.6	114/137	80.3
mterf22-3 homozygous line	157/161	97.5	88/102	86.3

*1 – A T-DNA insertional line mutant in *nMAT1* (At1g30010) gene (see Keren *et al*. 2012).
