# Supplementary figures and images for: Control of organelle gene expression by the mitochondrial transcription termination factor mTERF22 in Arabidopsis thaliana plants
Source: PLoS One. 2018 Jul 30;13(7):e0201631. doi: 10.1371/journal.pone.0201631 (PMC6066234; doi:10.1371/journal.pone.0201631)

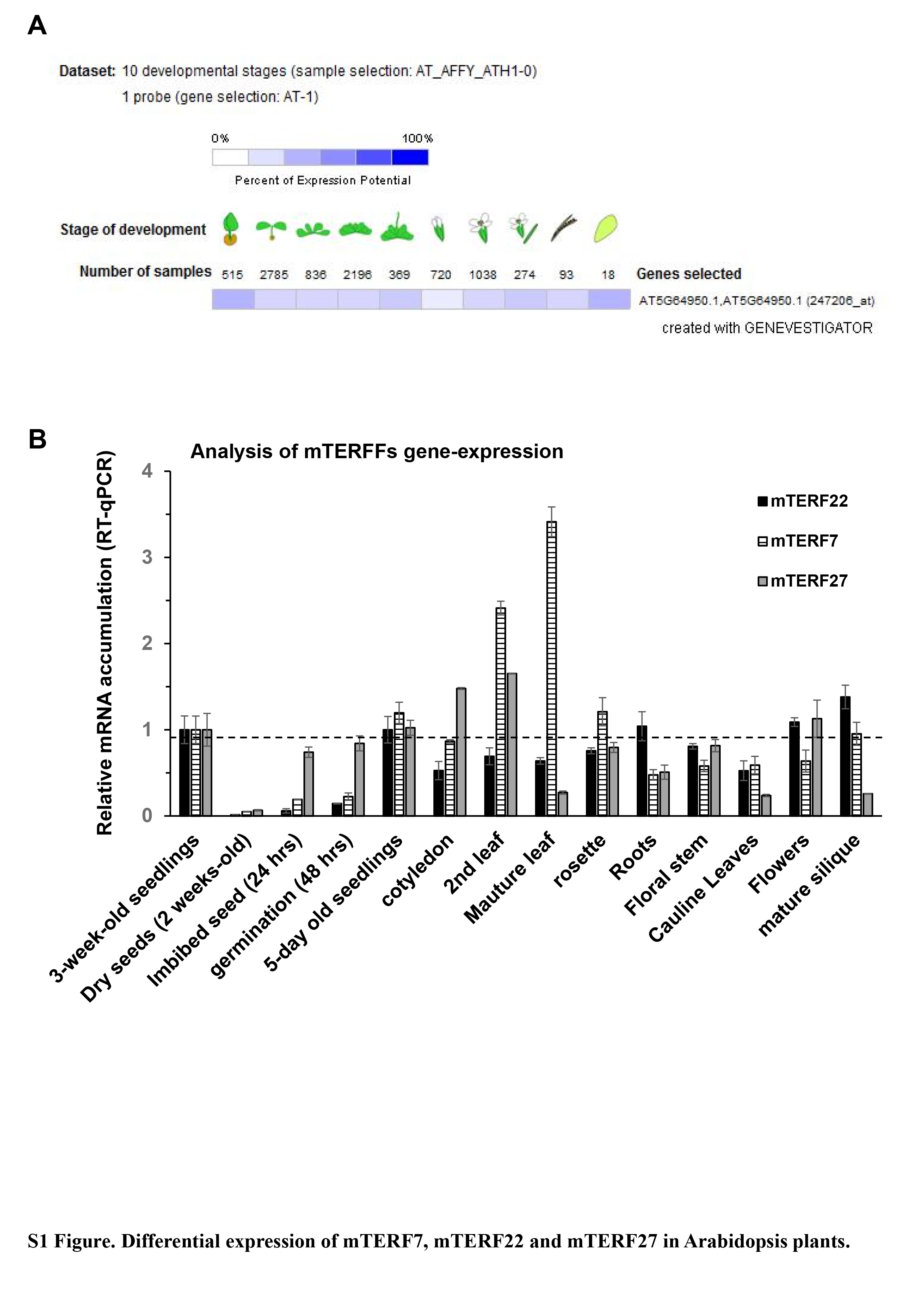

Supplement: S1 Fig — (A) Analysis of the expression profiles of mTERF22 (encoded by the At5g64950 gene-locus), available in the Genevestigator [65] and AtGeExpress [66] microarray databases, show that mTERF22 is expressed at low levels in different tissues throughout the plant’s development. (B) Differential expression of mTERF7 (At5g07900), mTERF22 and mTERF27 (At1g21150) in Arabidopsis thaliana (Col-0) plants. The relative steady-state levels of mRNAs corresponding to mTERF7, mTERF22 and mTERF27 was determined by RT-qPCR in 3 week-old wild-type, mterf22-1 and mterf22-2 plants after normalization to the actin2 (At3g1878) and 18S rRNA (At3g41768) genes (see S1 Table) [59–61, 63, 81]. The values are mean of three independent biological replicates, using 35~50 seedlings from each line in each assay. Error bars indicate one standard deviation). (TIF) [file pone.0201631.s005.tif]

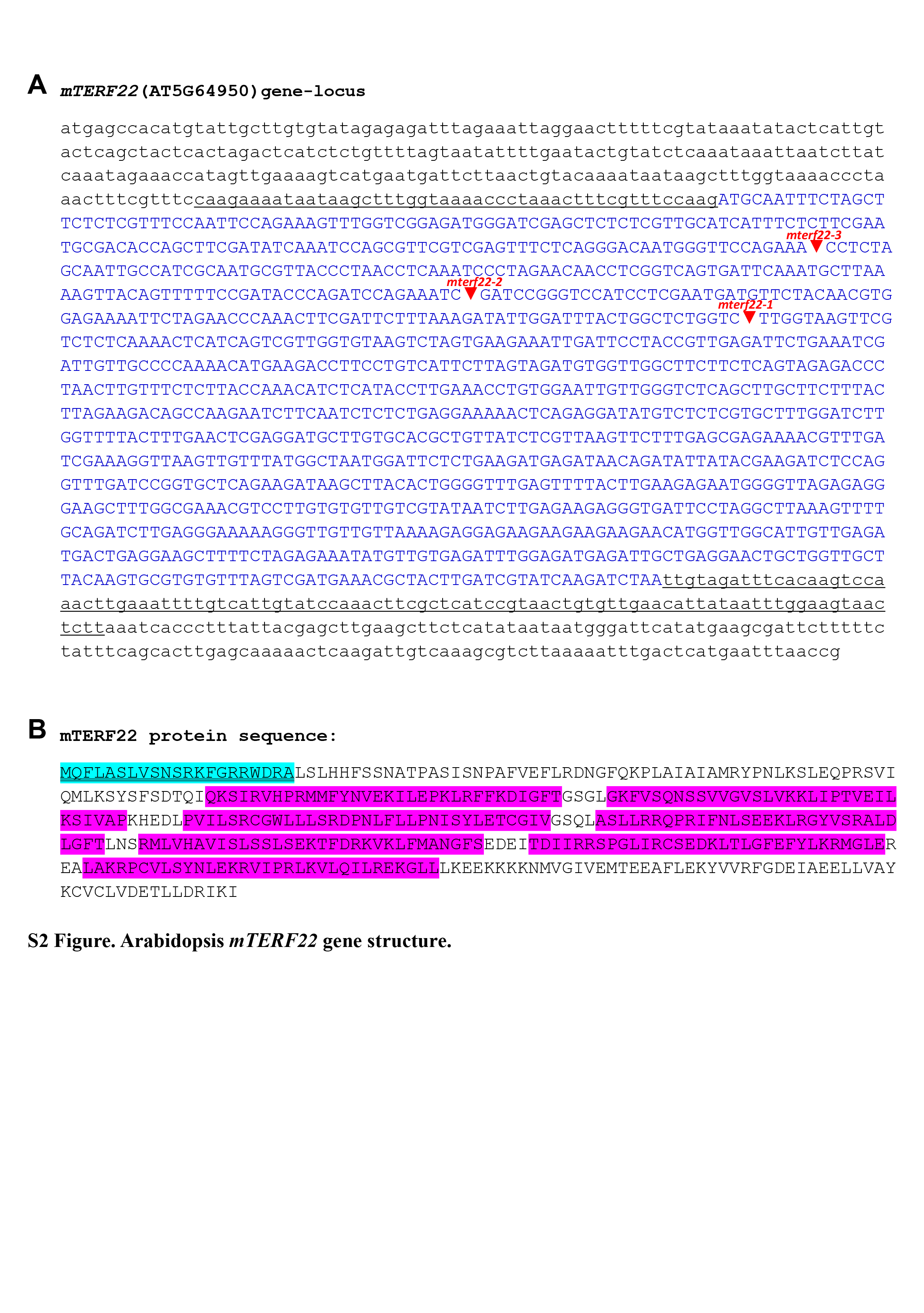

Supplement: S2 Fig — The nucleotides (A) and amino acids (B) sequences of mTERF22. Underlined letters indicate to the 5’ and 3’ untranslated regions (UTRs), as indicated by the RACE analysis and TAIR database, while uppercased letters represent the open reading frame of mTERF22. The position of T-DNA insertions in mterf22 mutants i.e., mterf22-1 (SALK- 032680), mterf22-2 (SAIL-1228) and mterf22-3 (SALK-133048) are indicated by red triangles. Panel B represents the amino acid sequence of Arabidopsis mTERF22 protein. The postulated regions corresponding to the mitochondrial targeting sequence (21 amino acid long, underlined and highlighted in blue) and the seven MTERF motifs (highlighted in magenta) of mTERF22 were predicted by the TargetP and SMART servers. (TIF) [file pone.0201631.s006.tif]

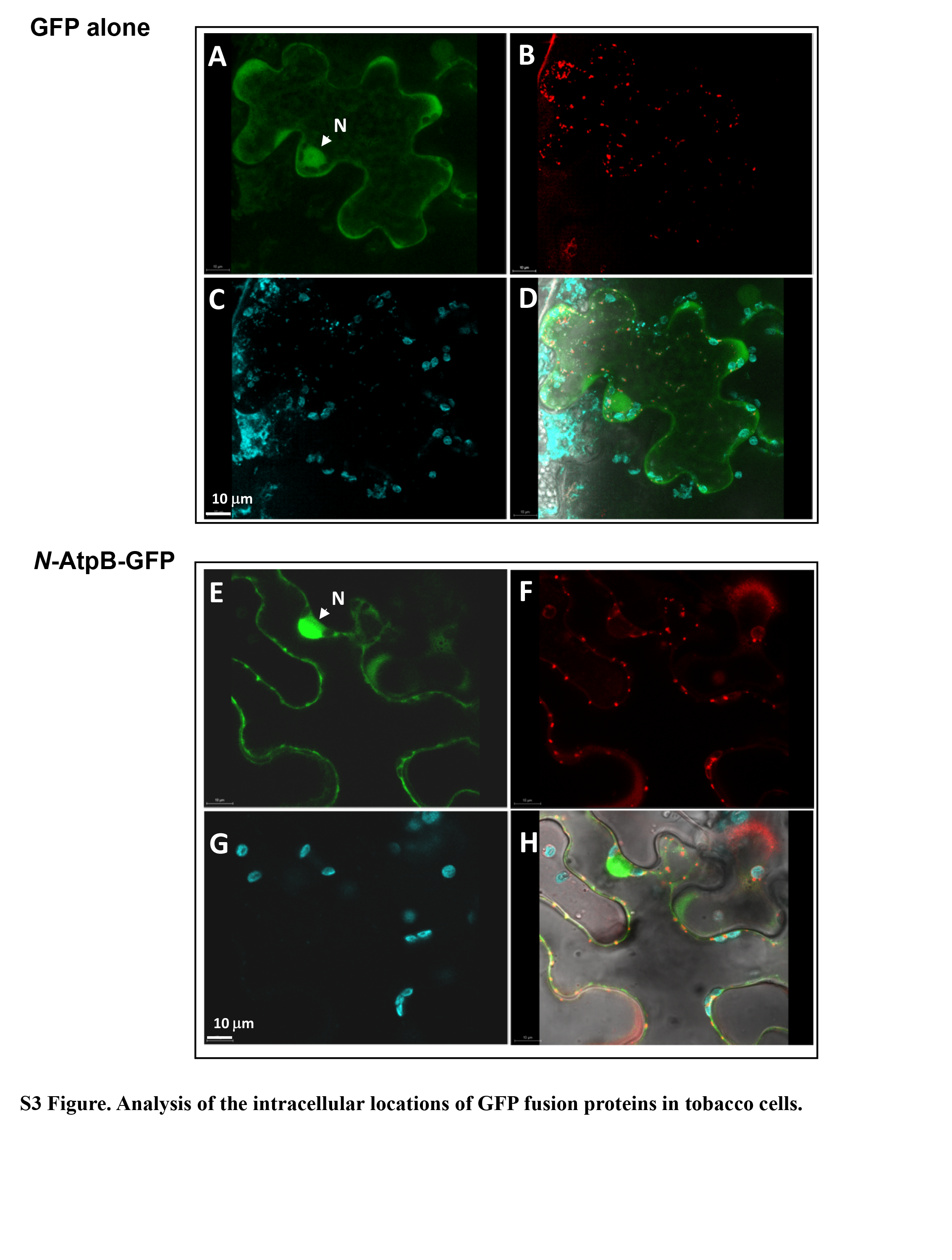

Supplement: S3 Fig — Tobacco plants were transformed with GFP alone (panels A to D) or GFP fused to the N-termini region (about 150 amino acids) of ATP synthase b-subunit (panels E to H). GFP signals (green, upper left, panels A and E), MitoTracker marker (red, upper right, panels B and F), chlorophyll autofluorescence (blue, lower left, panels C and G) and merged images (lower right, panels D and H), are outlined in each panel. The position of the nucleus (N) is indicated in panels A and E. (TIF) [file pone.0201631.s007.tif]

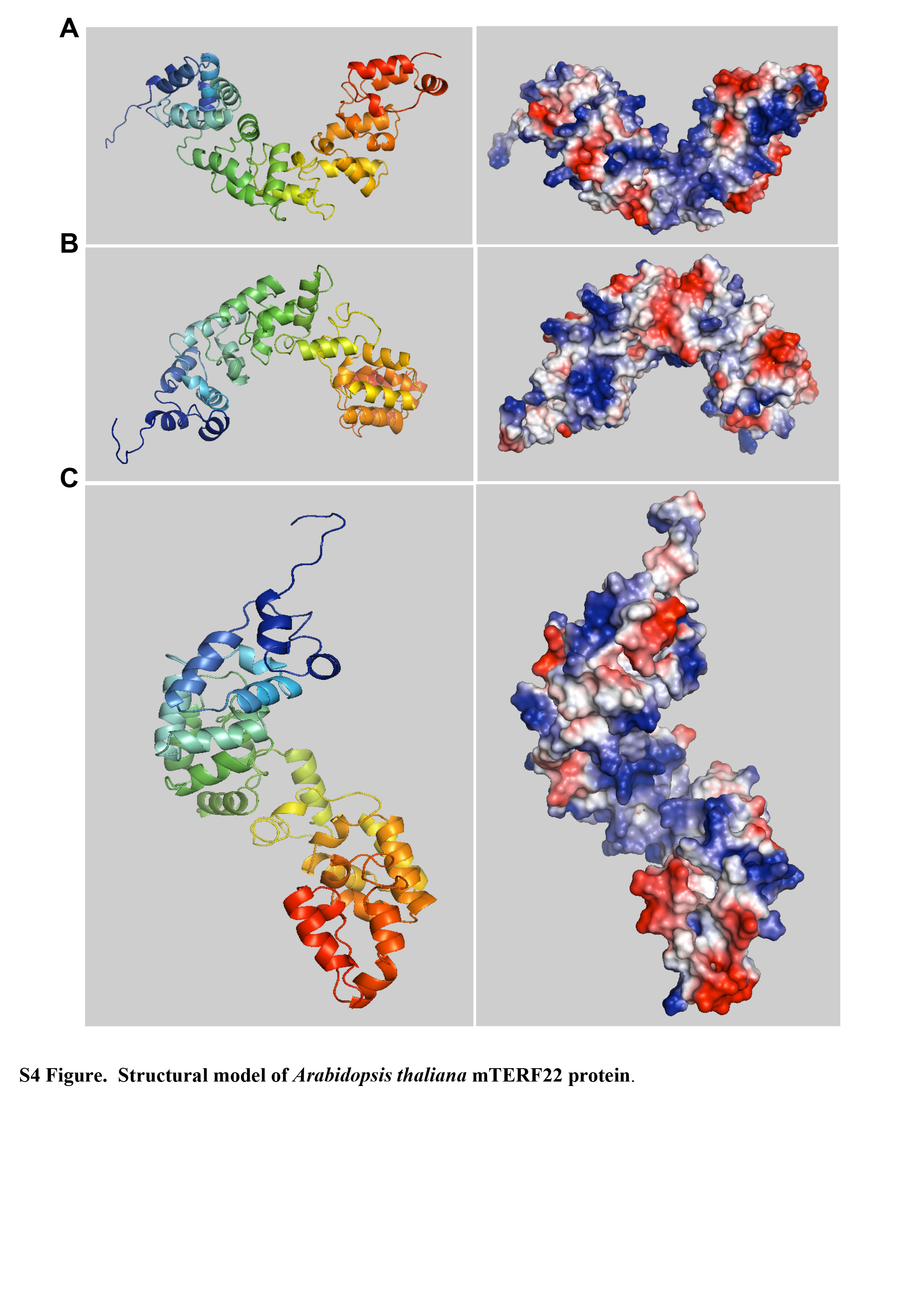

Supplement: S4 Fig — Schematic representation of the putative 3D structure of Arabidopsis mTERF22 protein. To get more of an insight on mTERF22's mode of action, in particular of DNA recognition, we performed an atomic model of the protein using the Phyre server (Kelley and Sternberg 2009). The model structure of mTERF22 (i.e. ribbon and surface views) were generated by the PyMol software suite [77]. A, B and C represent the same structure from different angles. Similarly to the mammalian mTERF1 and mTERF3 proteins [74–76], the predicted 3D structure of mTERF22 suggested a solenoid-like fold [103]. The color code is red for negative values, white for near zero values, and blue for positive values. Positively charged surfaces are expected to be critical for nucleotide recognition and binding, while uncharged or positively charged regions may function in protein-protein interactions. (TIF) [file pone.0201631.s008.tif]

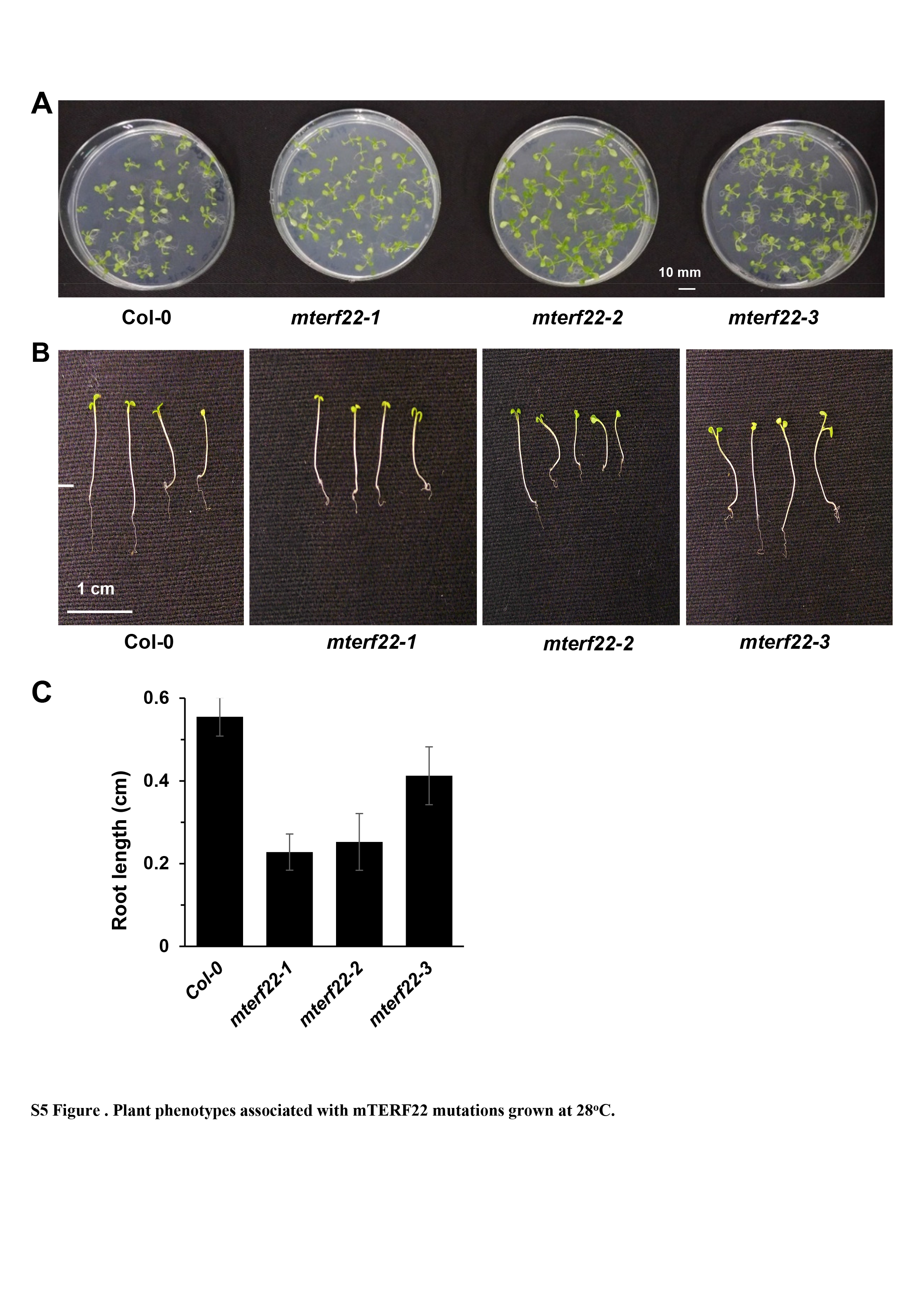

Supplement: S5 Fig — The effects of mTERF22-suppression on the growth phenotypes and development of Arabidopsis wild-type (Col-0) and mterf22 knockout lines. (A) Growth phenotypes associated with 3-week-old wild-type and homozygous mterf22 seedlings grown on MS-agar plates at 28°C. (B) 2-week-old wild-type and mterf22 seedlings grown vertically on MS-agar plates at 28°C. (C) The average root lengths of wild-type and mterf22 mutants grown at 28°C. The values are means of three biological replicates with ~30 seedlings from each line. Error bars indicate one standard deviation. Statistical significance was set at P < 0.05. (TIF) [file pone.0201631.s009.tif]

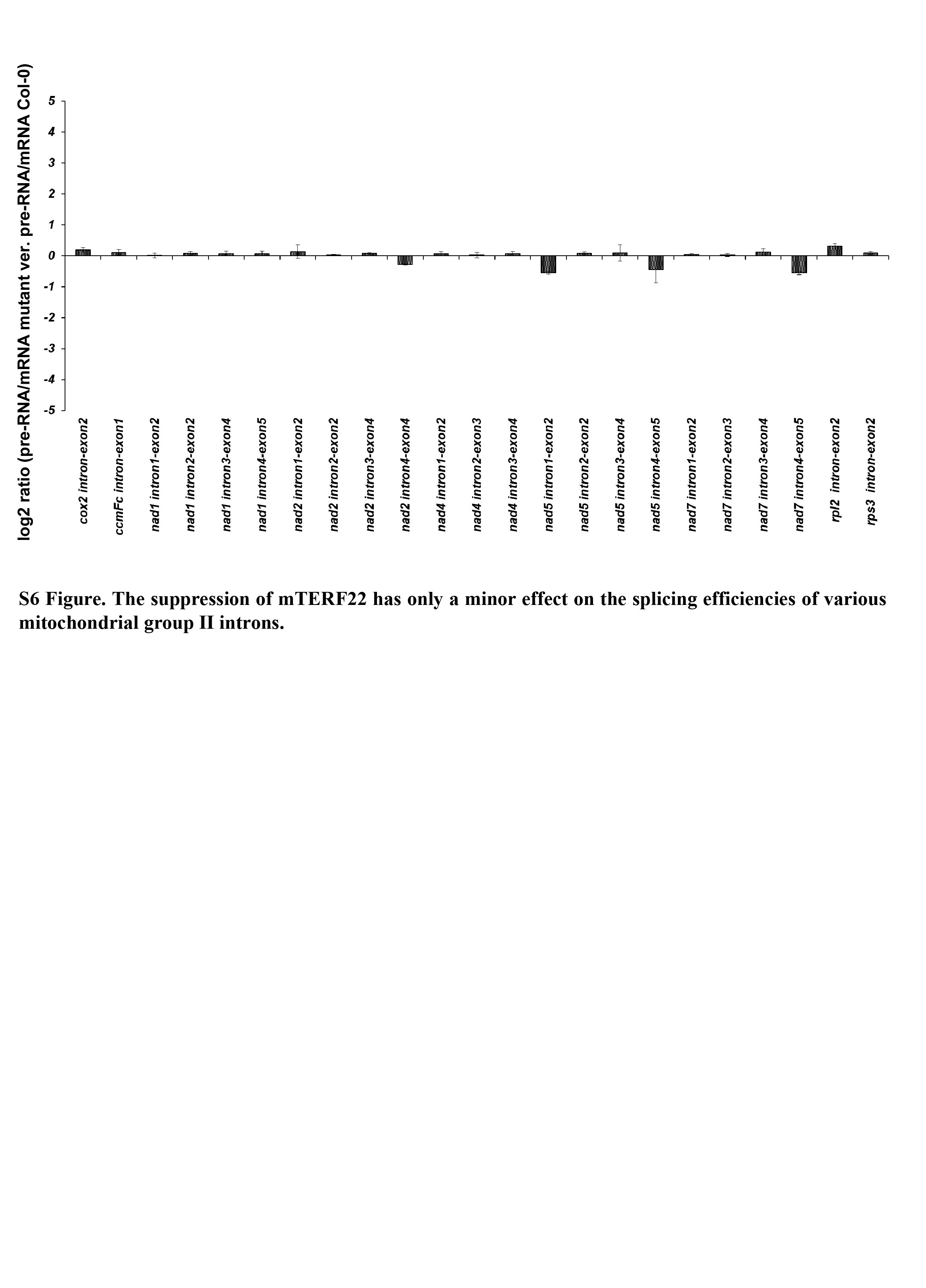

Supplement: S6 Fig — Quantitative RT-PCR of unspliced (pre-mRNA) and spliced (mRNA) mitochondrial transcripts in wild-type and mterf22-1 plants, was preformed as described in Zmudjak et al. (2017), after normalization to the actin2 (At3g1878), and 18S rRNA (At3g41768) genes. The histogram shows the ratios of pre-RNAs to mRNA between mterf22 and wild-type plants. The values are means of four biological replicates using 35~50 seedlings from each line in each assay. Error bars indicate one standard deviation. (TIF) [file pone.0201631.s010.tif]

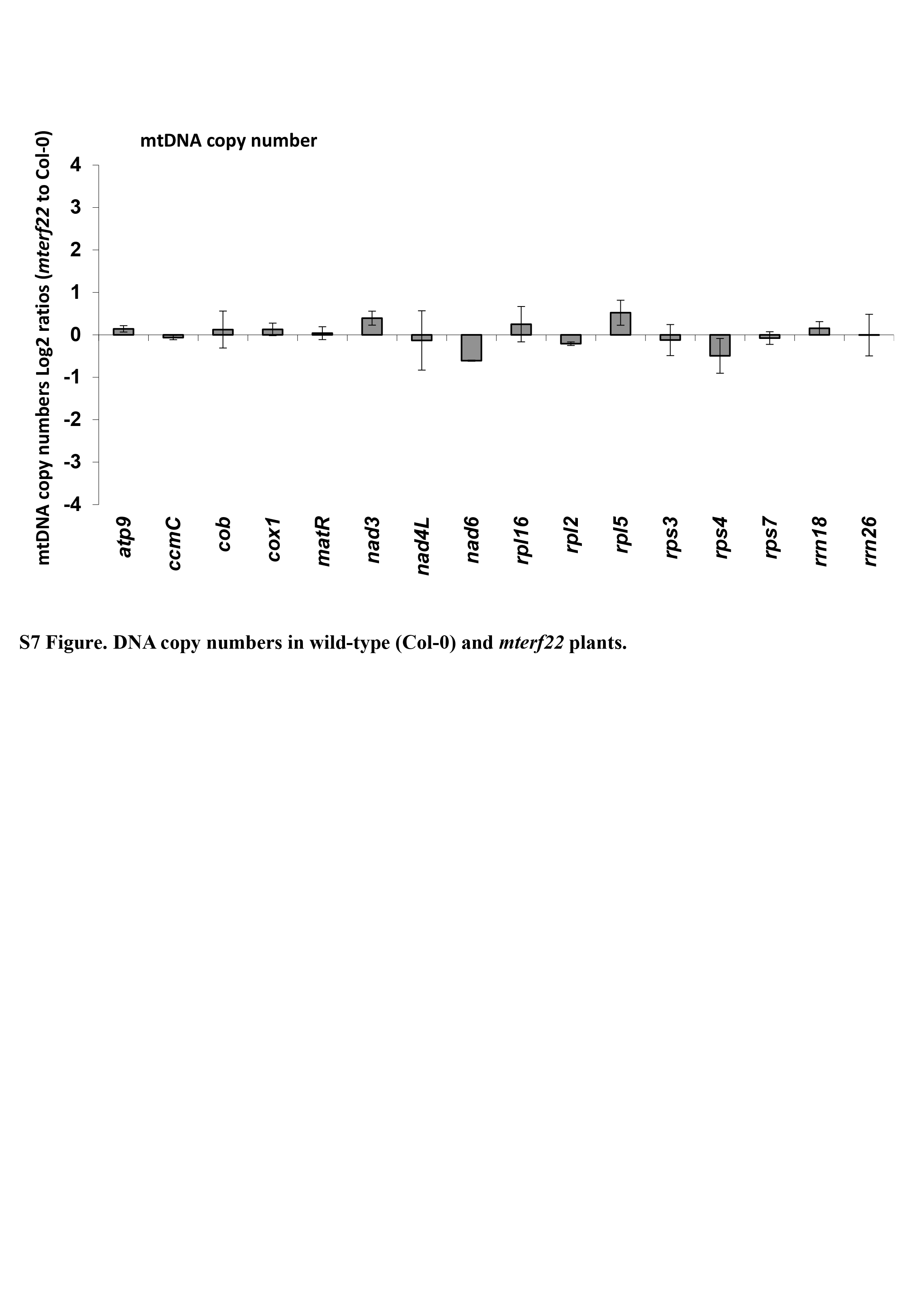

Supplement: S7 Fig — Relative mtDNA copy numbers in mterf22-1 mutants versus wild-type plants were analyzed by qPCR with oligonucleotides designed to different mitochondrial genes. Primers used in the qPCR analyses are listed in Supplemental S4 Table. The values are means of five biological replicates, using 35~50 seedlings from each line in each assay. Error bars indicate one standard deviation. (TIF) [file pone.0201631.s011.tif]

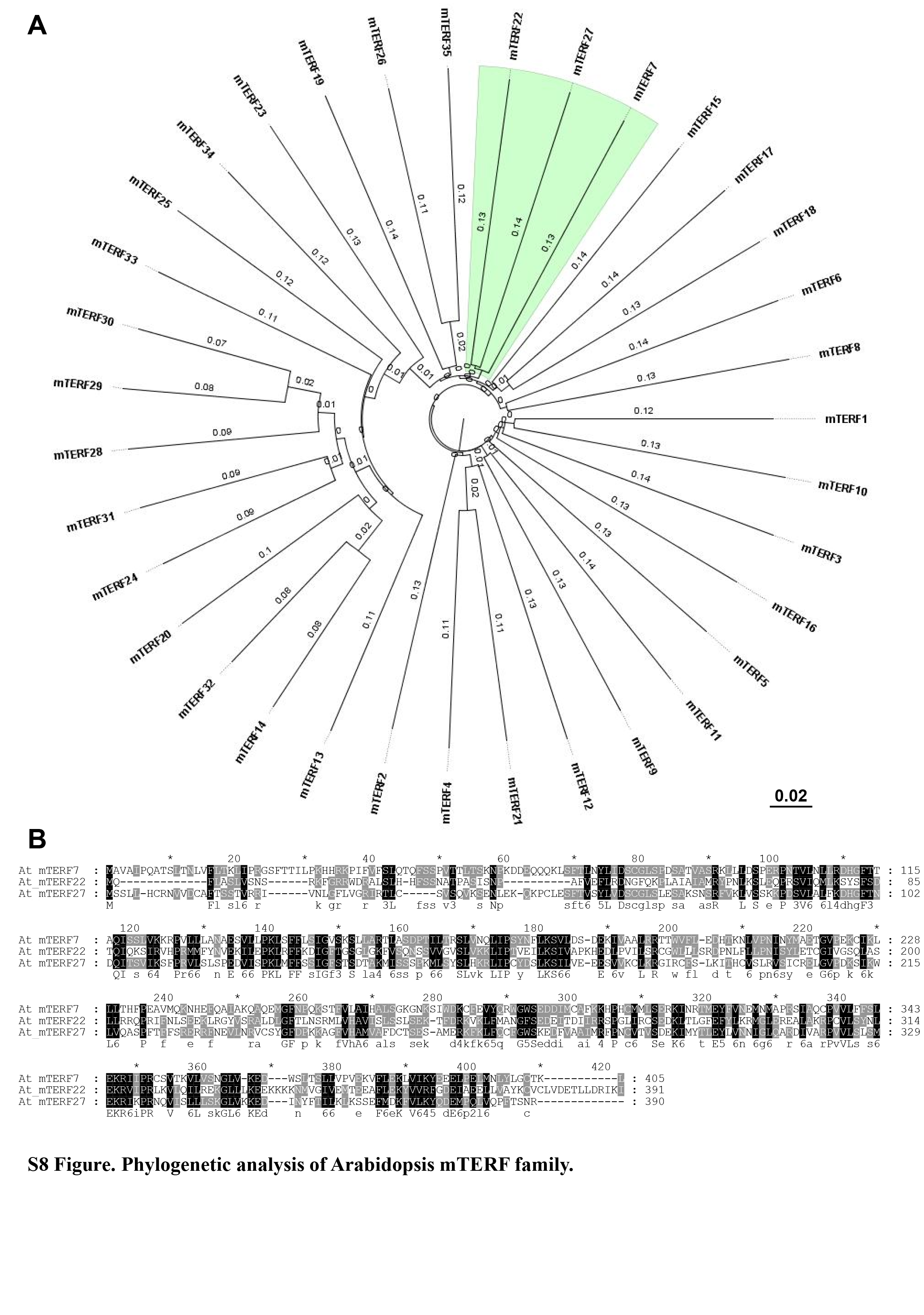

Supplement: S8 Fig — (A) Phylogeny tree was constructed using the T-Coffee multiple sequence alignment server [71], with the 35 known Arabidopsis mTERF protein sequences (bootstrap values for 1,000 bootstrap replicates). The scale bar represents the number of amino acid substitutions per site. (B) Alignment of mTERF7, mTERF22 and mTERF27 was conducted with T-Coffee multiple sequence alignment server, and displayed using GeneDoc [72] with the conserved residue shading mode. (TIF) [file pone.0201631.s012.tif]

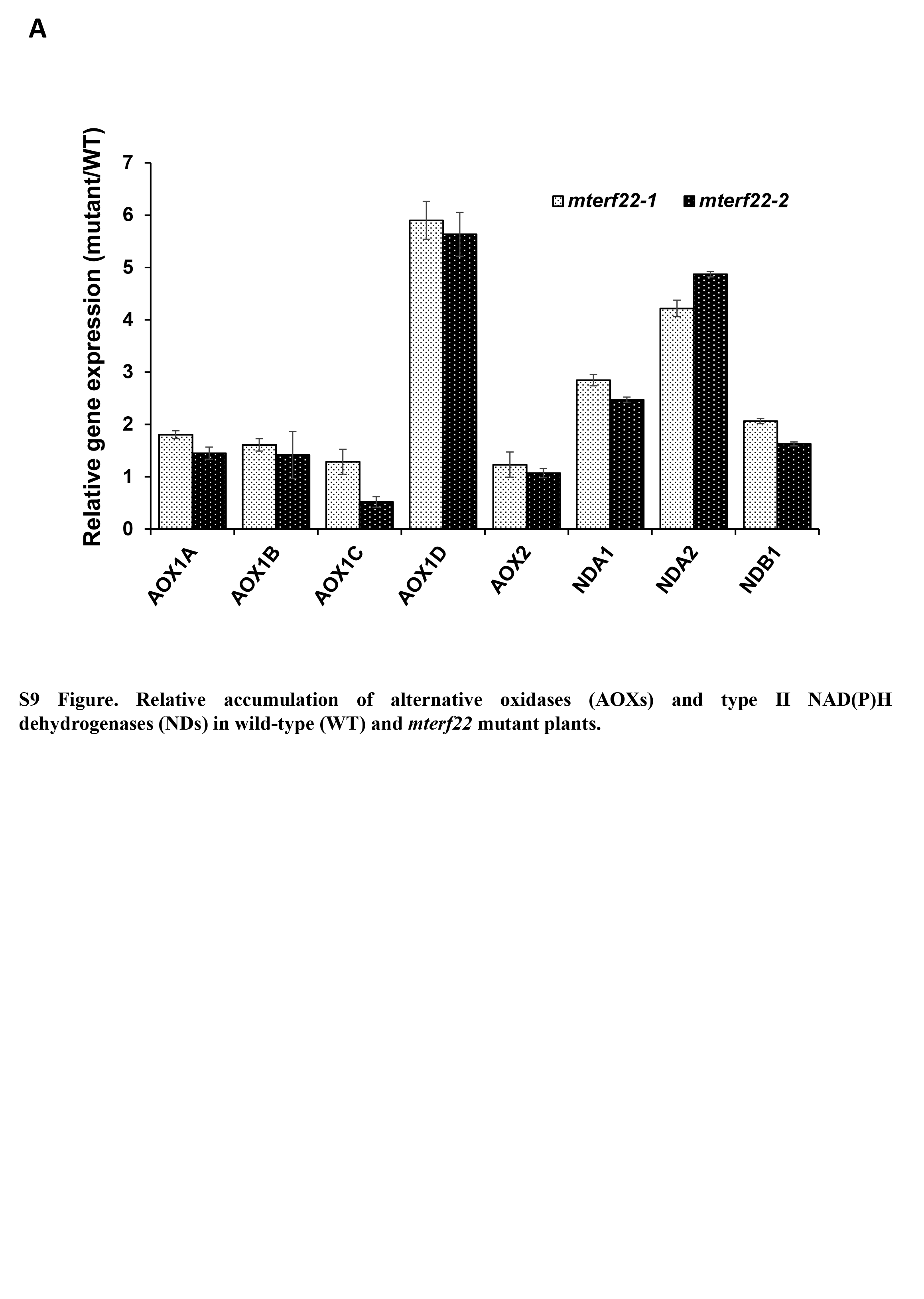

Supplement: S9 Fig — Relative quantification of nuclear-encoded genes related to the alternative respiration pathway in the wild type and mterf22 mutants. The relative steady-state levels of mRNAs corresponding to various AOXs and alternative NAD(P)H isoforms was determined by RT-qPCR in 3 week-old wild-type, mterf22-1 and mterf22-2 plants after normalization to the actin2 (At3g1878) and 18S rRNA (At3g41768) genes [59–61, 63, 81]. The values are mean of three independent biological replicates, using 35~50 seedlings from each line in each assay. Error bars indicate one standard deviation. (TIF) [file pone.0201631.s013.tif]

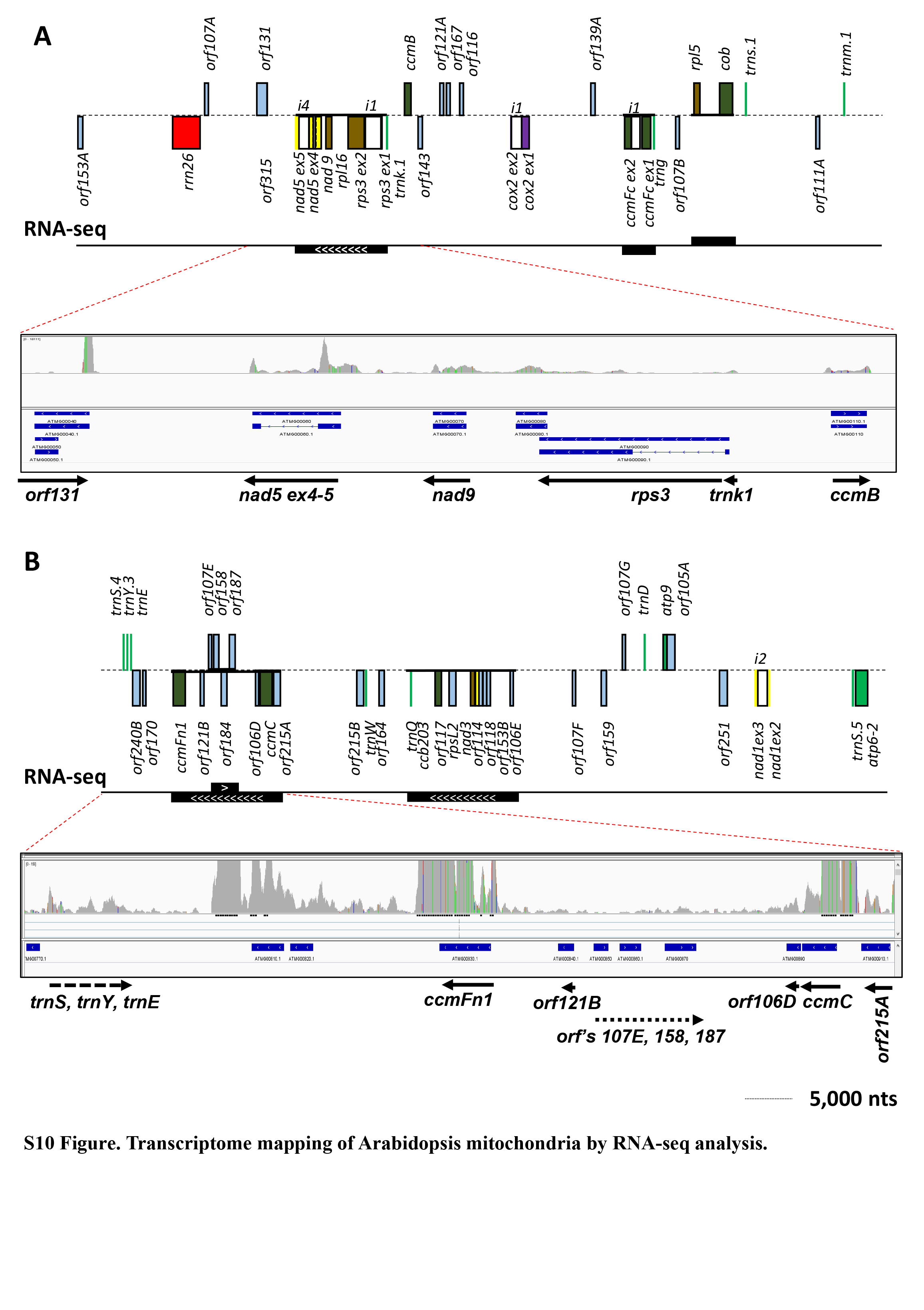

Supplement: S10 Fig — Total mtRNA was extracted from Arabidopsis mitochondria. RNA-sequencing (RNA-seq) was carried out on Illumina Genome Analyzer (The Genome Laboratory, The Hebrew University, Jerusalem, Israel), essentially as described previously [7, 61]. Data are shown for the regions encompassing the supposed polycistronic units of nad5-nad9-rps3 (A) and ccmC-ccmFn1 (B) gene clusters. Green, blue and red lines point to sequence variations (typically C-to-U RNA editing) between the mtRNA-seq data and the mtDNA of Arabidopsis (NC_001284; [84]). Black arrows indicate to the direction of transcription. (TIF) [file pone.0201631.s014.tif]

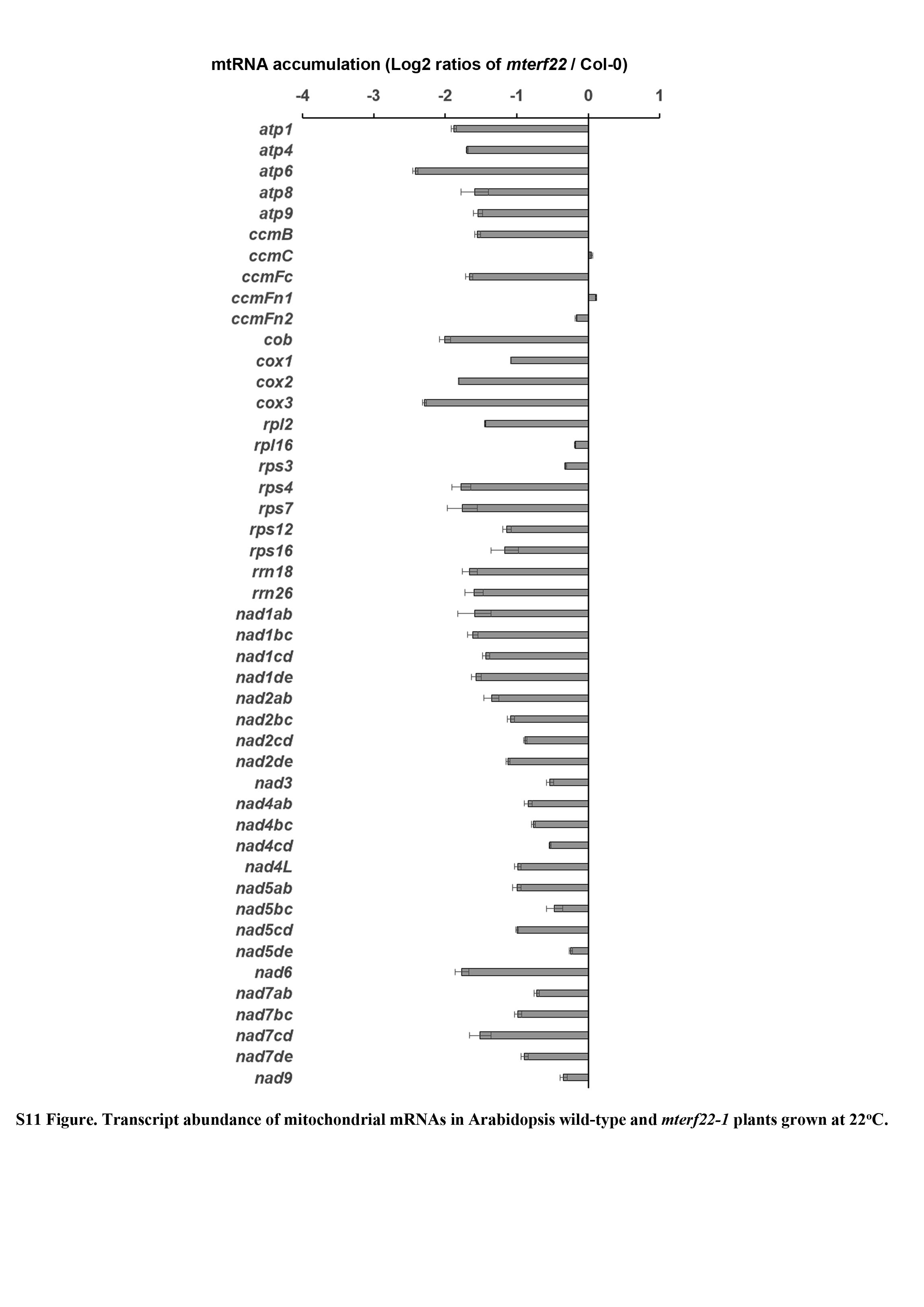

Supplement: S11 Fig — The accumulation of various mtRNAs in wild-type and mterf22 plants was analyzed by quantitative reverse transcription PCR (RT-qPCR). RNA extracted from 3-week-old wild-type (Col-0) and mterf22-1 mutant plants grown at 22°C was reverse-transcribed, and the relative steady-state levels of different organellar transcripts were evaluated by qPCR with specific oligonucleotides, after normalization to the actin2 (At3g1878), 18S rRNA (At3g41768) and 26S rRNA (Atmg00020) genes (see S1 and S3 Tables). The histogram shows the relative mRNAs levels (i.e. log2 ratios) in mutant lines versus those of wild-type plants. The values are means of three biological replicates, using about 35~50 seedlings from each line in each assay. Error bars indicate one standard deviation. (TIF) [file pone.0201631.s015.tif]

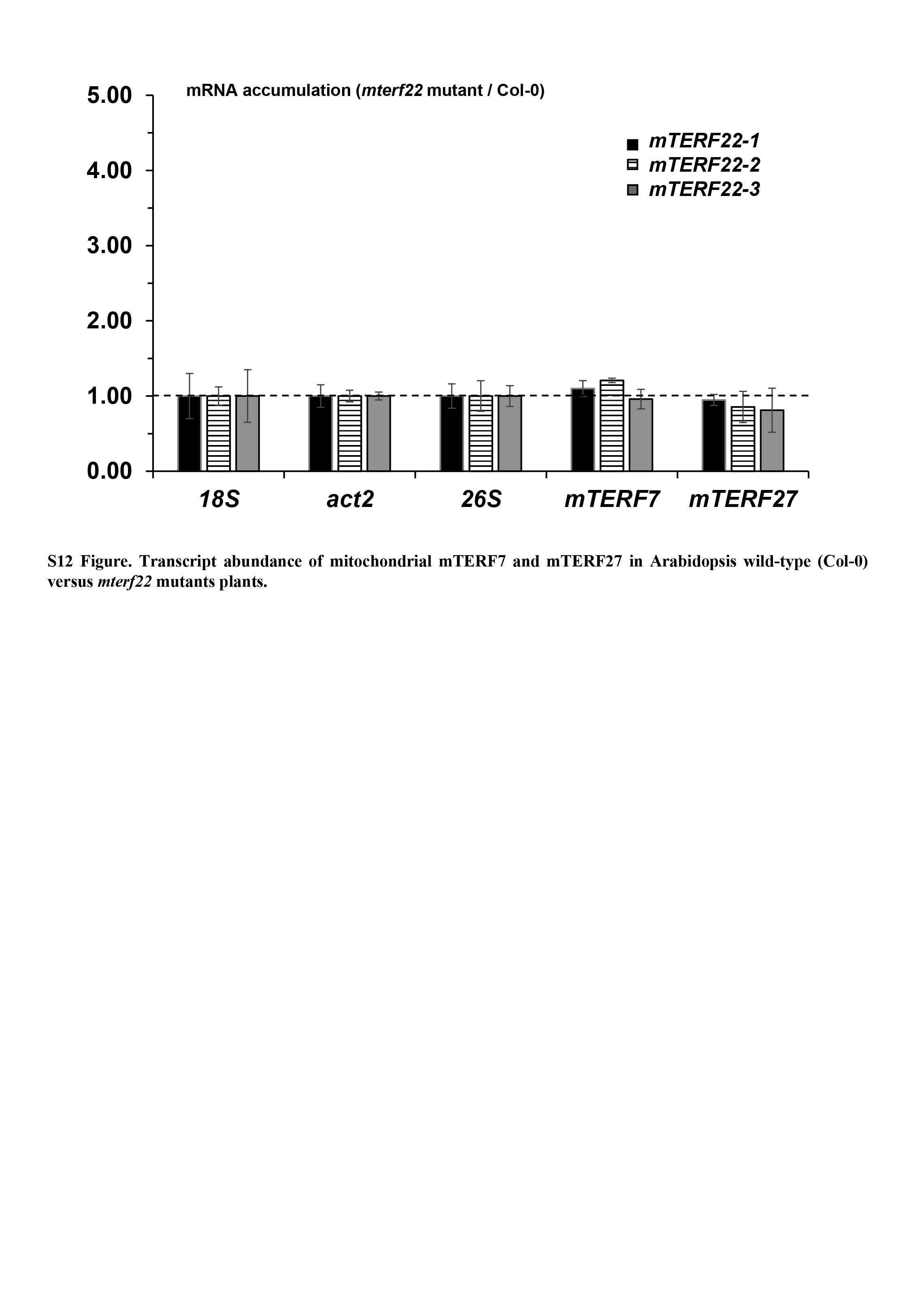

Supplement: S12 Fig — The accumulation of transcripts corresponding to mTERF7 and mTERF27 in wild-type (Col-0) and mterf22 plants was analyzed by quantitative reverse transcription PCR (RT-qPCR). RNA extracted from 3-week-old wild-type (Col-0) and mterf22 mutants plants grown at 22°C was reverse-transcribed, and the relative abundances (i.e., steady-state levels) of mTERF7 (AT5G07900) and mTERF27 (AT1G21150) transcripts were evaluated by qPCR with specific oligonucleotides, after normalization to the actin2 (At3g1878), 18S rRNA (At3g41768) and 26S rRNA (Atmg00020) genes (see S1 Table). The histogram shows the relative mRNAs levels in mutant lines versus those of the wild-type plants. The values are means of three biological replicates, using about 30 Arabidopsis seedlings. Error bars indicate one standard deviation. (TIF) [file pone.0201631.s016.tif]
